# *Garcinia kola*: a critical review on chemistry and pharmacology of an important West African medicinal plant

**DOI:** 10.1007/s11101-023-09869-w

**Published:** 2023-05-23

**Authors:** Jan Tauchen, Adela Frankova, Anna Manourova, Irena Valterova, Bohdan Lojka, Olga Leuner

**Affiliations:** 1 Department of Food Science, Faculty of Agrobiology, Food and Natural Resources, Czech University of Life Sciences, Prague, Czech Republic; 2 Department of Crop Sciences and Agroforestry, Faculty of Tropical AgriSciences, Czech University of Life Sciences, Prague, Czech Republic; 3 Institute of Organic Chemistry and Biochemistry, Academy of Sciences of the Czech Republic, Prague, Czech Republic

**Keywords:** Kolaviron, Medicinal potential, Traditional medicine, Flavonoids, Agroforestry

## Abstract

*Garcinia kola* Heckel (Clusiaceae) is a tree indigenous to West and Central Africa. All plant parts, but especially the seeds, are of value in local folklore medicine. *Garcinia kola* is used in treatment of numerous diseases, including gastric disorders, bronchial diseases, fever, malaria and is used to induce a stimulating and aphrodisiac effect. The plant is now attracting considerable interest as a possible source of pharmaceutically important drugs. Several different classes of compounds such as biflavonoids, benzophenones, benzofurans, benzopyran, vitamin E derivatives, xanthones, and phytosterols, have been isolated from *G. kola*, of which many appears to be found only in this species, such as garcinianin (found in seeds and roots), kolanone (fruit pulp, seeds, roots), gakolanone (stem bark), garcinoic acid, garcinal (both in seeds), garcifuran A and B, and garcipyran (all in roots). They showed a wide range of pharmacological activities (e.g. analgesic, anticancer, antidiabetic, anti-inflammatory, antimalarial, antimicrobial, hepatoprotective and neuroprotective effects), though this has only been confirmed in animal models. Kolaviron is the most studied compound and is perceived by many studies as the active principle of *G. kola*. However, its research is associated with significant flaws (e.g. too high doses tested, inappropriate positive control). Garcinol has been tested under better conditions and is perhaps showing more promising results and should attract deeper research interest (especially in the area of anticancer, antimicrobial, and neuroprotective activity). Human clinical trials and mechanism-of-action studies must be carried out to verify whether any of the compounds present in *G. kola* may be used as a lead in the drug development.

## Introduction

*Garcinia kola* Heckel (Clusiaceae), a multipurpose tree commonly found in subtropical and tropical moist lowland forests of Nigeria, Cameroon and other countries in sub-Saharan Africa. It is colloquially called bitter kola, false kola or sometimes “wonder plant” because almost every part of this tree has been used in traditional medicine for broad portfolio of ailments since ancient times (Ijomone and Obi 2013; Maňourová et al. 2019; Erukainure et al. 2021). The seeds are highly valued as oral masticatory agent with bitter astringent taste and stimulant effect. They (as well as other plant parts) are used to treat wide range of diseases, including as gastric and liver disorders, diarrhoea, bronchial diseases, throat infections, colds, fever, malaria, and as an aphrodisiac (see Table 1) (Erukainure et al. 2021). Especially the use in the area of liver protection and disease, throat infection, colds, and the aphrodisiac action is often repeated in the literature. The seeds are habitually chewed as a part of traditional, cultural and social ceremonies and for their aphrodisiacal effect (Maňourová et al. 2019). It is often given to guests and unfamiliar persons as sign of friendship and respect. Currently, *G. kola* is recorded as “vulnerable” in IUCN’s Red List of Threatened Species*,* possibly due to deforestation practices and relatively intensive collection from the wild (Cheek 2004).

**Table 1:** Ethnomedicinal information of different plant parts of *G. kola*

Plant part	Ethnomedicinal use	Route of application	References
Root	For oral hygiene	Chew sticks	Uko et al. (2001), Okoye et al. (2014), Tcheghebe et al. (2016)
Stem bark	Purgative	N/A	
Latex from the bark	Treatment of inflammation and for healing of wounds	External application	
Mixture of leaves and bark	Hypertension, malaria, liver diseases, asthma and gastroenteritis	Infusion	Tcheghebe et al. (2016), Dogara et al. (2022)
Seeds	Bronchitis, laryngitis and throat infections	Raw seed chewed or processed into infusion	Kabangu et al. (1987), Iwu et al. (1999), Okoye et al. (2014), Tcheghebe et al. (2016), Teodoro et al. (2021)
	Treatment of head or chest colds and relieve of cough		
	Colic		
	Rheumatism		Tcheghebe et al. (2016)
	Menstrual cramps (analgesic activity)		
	As a stimulant to induce alertness and insomnia		Uko et al. (2001), Okoye et al. (2014)
	Aphrodisiac		
	Food and alcohol poisoning, and as a poison antidote		Kabangu et al. (1987), Braide (1991), Ikpesu et al. (2014), Tcheghebe et al. (2016)
	Liver disorders (liver protection)		Iwu et al. (1990b), Iwu et al. (2002), Tcheghebe et al. (2016), Teodoro et al. (2021)
	Diabetes		Teodoro et al. (2021), Dogara et al. (2022)
	Dysentery and diarrhoea		Ainslie (1937), Tcheghebe et al. (2016)
	As an antimicrobial, antiviral, and antiparasitic agent		Iwu et al. (2002), Okoye et al. (2014), Kluge et al. (2016)
	Induction of wound healing	Oil pressed form the seeds used externally	Tcheghebe et al. (2016)

*N/A* information not available

Over the last few years, *G. kola* has received quite large research attention, mainly due to content of a very specific biflavonoid complex collectively referred to as kolaviron, whose distribution seems to be limited to *G. kola*. More recently, this research attention has resulted in emergence of a few review articles (Maňourová et al. 2019; Erukainure et al. 2021; Dogara et al. 2022; Emmanuel et al. 2022) that introduce *G. kola*, and kolaviron, as a promising material for drug discovery. However, kolaviron is not the only constituent found in *G. kola* and it contains other compounds (e.g. garcinianin, kolanone, gakolanone, garcinoic acid, garcinal, garcifuran A and B, and garcipyran) (Hussain et al. 1982; Niwa et al. 1993, 1994a, b; Terashima et al. 1995, 1997; Akoro et al. 2020) that also appears to be very specific for *G. kola* and their presence have thus fur not been confirmed in any other botanical source. These compounds are to a very large extent neglected in these review articles and kolaviron is perceived as the active principle, though these lesser-known compounds may provide interesting pharmacological activities as well. On top of that, kolaviron is in majority of available studies (animal models) tested in very large doses, which appears rather unrealistically high and untransferable to clinical practice. Clinical data on humans on any of the constituent found in *G. kola* are entirely missing. Despite of this fact, these reviews draw conclusions on therapeutic efficacy of *G. kola* and kolaviron. In view of what is written above, this review offers a critical update on available information of the most studied and discussed compound of *G. kola*, kolaviron, and provides analysis of existing knowledge on other present constituents.

## Methodology and search strategy

The information summarized in this review was obtained through extensive literature review and search of relevant books and articles with the use of Web of Knowledge, SciVerse Scopus and PubMed databases. The search was conducted during the period of 2020–2022 (search period: 1967–2022), using specific keywords, including: “garcinia kola” (no. of hits ≈ 500), “kolaviron” (188), “kolaflavanone” (20), “garcinianin” (5), “amentoflavone” (1021), “volkensiflavone” (51), “morelloflavone” (114), “fukugetin” (36), “kolanone” (6), “gakolanone” (1), “garcinol” (399), “garcionic acid” (29), “garcinal” (6), “garcifuran” (3), and “garcipyran” (1). Due to the absence of human clinical trials, studies based on both in vitro and in vivo conditions were included in the review, however, only those studies that used isolated substances (studies using extracts were excluded from the selection). The objective of this review is to present a comprehensive summary of all scientifically accessible information on the chemical composition and reported biological activities of isolated compounds present in *G. kola* and critically assess if they may indeed be of value in clinical practice.

## Results

### Chemical composition

#### Primary metabolites

Although *G. kola* seeds are more valued for their medicinal properties rather than as foodstuff, the kernels are still commonly consumed, which justifies concerns about their nutritional value (Okoye et al. 2014). There are wide discrepancies among the published results on the species primary metabolites content. Generally, the studies agree on relatively high amounts of moisture in the seeds (about 70%), suggesting their vulnerability to mould infestation and possible storage/post-harvest processing difficulties. Present saccharides, also described as nitrogen-free extracts (NFE), form the largest part of the seed proximate composition (around 65%), while the content of minerals is very low (1.5% on average). The mean value for crude protein was found to be 3.5%, with lysine (2.4 g/kg), leucine (1.9 g/kg) and valine (1.7 g/kg) being the predominant essential amino acids (AA) and glutamic acid (6.8 g/kg) with arginine (5.5 g/kg) as the highest abundant nonessential AA in both kernels and seeds’ hulls (Eleyinmi et al. 2006). The crude fat generally varies about 6.2% with oleic acid (C 18:1; 38 mg/kg), linoleic acid (C 18:2; 36 mg/kg) and palmitic acid (C 16:0; 32 mg/kg) being the dominant fatty acids in both seeds and hulls (Eleyinmi et al. 2006). The crude fibre content was determined at 9.4% on average. Before consumption, people generally prefer to peel the seeds, discarding the hulls as worthless waste. However, due to their high protein content (9.92 g/100 g), these husks may represent a valuable fodder source for domestic animals, whose diet is usually based only on natural pastures of poor quality and thus quite low in protein content (Eleyinmi et al. 2006). If grinded into a powder, the hulls can be incorporated into enriched feeding mixtures.

Quite limited information is available on the micronutrient content of *G. kola* seeds. They were reported to contain relatively high amounts of vitamin C (23.1–69 mg/100 g), potassium (25–722 mg/kg) and phosphorus (3.3–720 mg/kg) (Okwu 2005; Onyekwelu et al. 2015). They are also low in anti-nutrients such as phytate and oxalate, and are thus considered safe for consumption without any reports on harmful overdosing (Onyekwelu et al. 2015; Konziase 2015).

#### Secondary metabolites

**Fig. 1:**
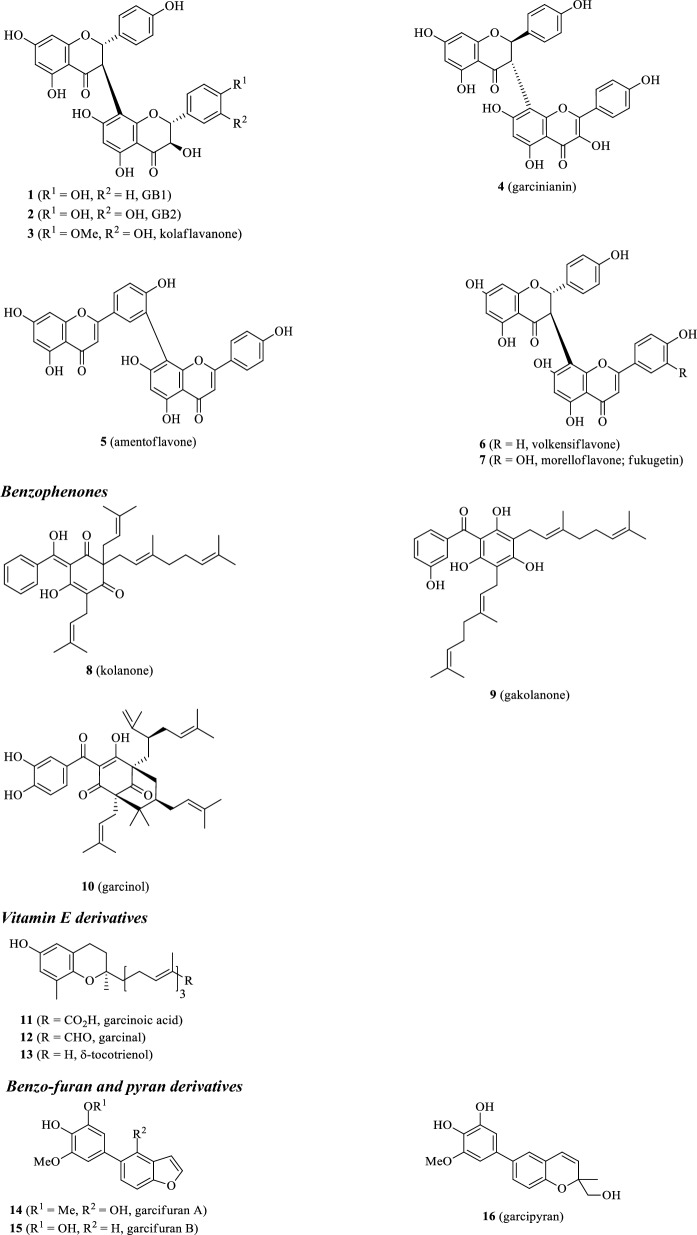

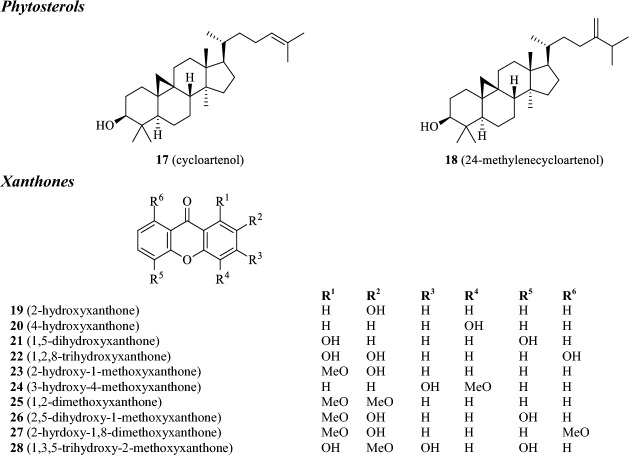
Chemical structures of secondary metabolites found in bitter kola (*Garcinia kola*)

Various classes of secondary metabolites have been isolated from different plant parts of *G. kola*. Of these, perhaps the most studied are flavonoids and their related structures. Benzophenone, benzofurans and benzopyran analogues, vitamin E derivatives, xanthones and phytosterols have also been isolated from *G. kola* in the past. Many of the present constituents, namely, kolaviron, garcinianin, kolanone, gakolanone, garcionic acid, garcinal, garcifuran A and B, and garcipyran A, appear to be exclusive for *G. kola* and have not been thus found in any other plant species yet. A list of known compounds isolated from *G. kola*, including plant parts where these constituents have been found, are given in Table 2. Their corresponding structures are illustrated in Fig. 1.

**Table 2:** Secondary metabolites found in bitter kola (*Garcinia kola*)

Compound		Plant part(s)	References
*Flavonoid structures*			
1	GB1*	Seeds, roots	Iwu et al. (1990c), Terashima et al. (1997), Erukainure et al. (2021)
2	GB2*	Seeds, roots	
3	Kolaflavanone*	Seeds, roots	
4	Garcinianin*	Seeds, roots	Terashima et al. (1995, 1997), Ajayi et al. (2014)
5	Amentoflavone	Seeds, wood	Iwu and Igboko (1982)
6	Volkensiflavone	Wood	Acuña et al. (2012)
7	Morelloflavone (fukugetin)	Wood	
*Benzophenones*			
8	Kolanone*	Fruit pulp, seeds, roots	Hussain et al. (1982), Iwu et al. (1990c), Madubunyi (1995)
9	Gakolanone*	Stem bark	Akoro et al. (2020)
10	Garcinol	Roots	Niwa et al. (1993)
*Vitamin E derivatives*			
11	Garcinoic acid*	Seeds	Terashima et al. (1997)
12	Garcinal*	Seeds	
13	δ-tocotrienol	Seeds	
*Benzo-furan and -pyran analogues*			
14	Garcifuran A*	Roots	Niwa et al. (1994b)
15	Garcifuran B*	Roots	
16	Garcipyran*	Roots	Niwa et al. (1994a)
*Phytosterols*			
17	Cycloartenol	Roots	Iwu et al. (1990c)
18	24-methylenecycloartenol	Roots	
*Xanthones*			
19	2-hydroxyxanthone	Stem	Terashima et al. (1999)
20	4-hydroxyxanthone	Stem	
21	1,5-dihydroxyxanthone	Stem	
22	2-hydroxy-1-methoxyxanthone	Stem	
23	3-hydroxy-4-methoxyxanthone	Stem	
24	1,2-dimethoxyxanthone	Stem	
25	2,5-dihydroxy-1-methoxyxanthone	Stem	
27	2-hyrdoxy-1,8-dimethoxyxanthone	Stem	
28	1,3,5-trihydroxy-2-methoxyxanthone	Stem	

*Compounds thus far only found in *G. kola*

At least seven biflavonoid structures have been characterized in *G. kola*, the most known and studied being kolaviron. Kolaviron is the principal biflavonoid mixture in the seeds and constitutes of biflavonoids GB1 (**1**), GB2 (**2**), and kolaflavone (**3**) (Ijomone and Obi 2013). Some authors confirmed the presence of kolavirone in the roots as well (Iwu et al. 1990c). Seeds and roots were also found to contain garcinianin (**4**) (Terashima et al. 1995; Ajayi et al. 2014). It appears that both kolaviron and garcinianin are exclusively produced by *G. kola* and are not found in other *Garcinia* species. *Garcinia kola* seed also contains amentoflavone (**5**) (Iwu and Igboko 1982); which is quite abundantly distributed across plant species (e.g. *Gingko biloba* and *Hypericum perforatum*) (Lobstein-Guth et al. 1988; Baureithel et al. 1997). Other biflavonoids occurring in *G. kola* include volkensiflavone (**6**) and morelloflavone (fukugetin; **7**); so far they were only identified in the wood (Acuña et al. 2012). On the other hand, both compounds were also discovered in fruits of other *Garcinia* species (e.g. *G. spicata*, *G. xanthochymus*, *G. intermedia*, *G. livingstonei*, *G. hombroniana*), suggesting that they also occur in the fruits and seeds of *G. kola*. The benzophenones in *G. kola* are represented by kolanone (**8**), gakolanone (**9**), and garcinol (**10**). Kolanone was the first discovered benzophenone derivative in *G. kola*. It was found in various plant parts, including the fruit (Hussain et al. 1982), seeds (Madubunyi 1995; Uwagie-Ero et al. 2020), and roots (Iwu et al. 1990c). As with biflavonoid kolavirone, the distribution of kolanone appears to be limited to *G. kola* and its presence has not yet been demonstrated in any other species. One recent study also confirms occurrence of structurally related gakolanone in *G. kola* stem bark (Akoro et al. 2020). It as well seems to be restricted only to *G. kola*. It was also discovered that the roots contain garcinol (Niwa et al. 1993). In comparison to kolanone and gakolanone, garcinol is widely distributed throughout the *Garcinia* species (including *G. indica*, *G. huillensis*, and *G. pedunculata*) (Kopytko et al. 2021). Additionally, it was discovered, that the seeds contain very specific derivatives of vitamin E, garcinoic acid (**11**) and garcinal (**12**) that appears to be also limited for *G. kola*. Along with these specific vitamin E analogues, δ-tocotrienol (**13**) has also been found in seeds (Terashima et al. 1997). Niwa et al. (1994a, b) have isolated two related benzofuran and one benzopyran derivatives, garcifuran A (**14**) and B (**15**), and garcipyran (**16**), from the roots. Again, all three compounds have so far only been found in *G. kola*, suggesting that this is their only-producing species. The roots were also found to contain cycloartenol (**17**) and 24-methylenecycloartenol (**18**). Several related xanthone analogues, namely 2-hydroxy-, 4-hydroxy-, 1,5-dihydoxy-, 1,2,8-trihydroxy-, 2-hydroxy-1-methoxy-, 3-hydroxy-4-methoxy-, 1,2-dimethoxy-, 2,5-dihydroxy-1-methoxy-, 2-hydroxy-1,8-dimethoxy-, and 1,3,5-trihydroxy-2-methoxy-xanthone (**19–28**) have been detected in the stems (Terashima et al. 1999). Both the cycloartenol and xanthone derivatives are quite abundant in the plant kingdom (El-Seedi et al. 2009; Gwatidzo et al. 2014). Some studies have indicated presence of a number of other compounds, including saponins, cardiac glycosides, alkaloids, and tannins (Adesuyi et al. 2012; Winner et al. 2016; Eleazu et al. 2012; Monago and Akhidue 2002). However, these studies only provide the total content of the given group of substances. It is worthy of note that there seems to be data on the total content only for seeds and leaves (for more details see Table 3). As far as we know, there is unfortunately a lack of studies providing concentrations of individual compounds. In addition to the substances discussed so far, *G. kola* seeds were also found to contain various cytochalasins (e.g. 8-metoxycytochalasin J, cytochalasin H and J, and alternariol) that appears not to be synthesized by the plant itself, but are the product of a plant-associated fungus of the genus *Phomopsis* sp. (Jouda et al. 2016).

**Table 3:** Amounts of given classes of compounds in *G. kola*

Plant part	Seed			Leaf
Unit	g/100 (dw)	g/100 (dw)*	mg/100 g (ww)	g/100 g (dw)
Saponins	2.47 ± 0.0	2.35 ± 0.16	15.79 ± 0.28	1.92 ± 0.82
(Cardiac) glycosides	3.42 ± 0.0	3.11 ± 0.20	67.10 ± 0.03	–
Alkaloids	0.65 ± 0.20	–	–	4.00 ± 0.21
Phenols	0.15 ± 0.00	–	–	
Flavonoids	2.04 ± 0.30	2.67 ± 0.54	–	1.10 ± 0.85
Tannins	0.34 ± 0.00	1.08 ± 0.10	0.69 ± 0.01	traces
References	Adesuyi et al. (2012)	Winner et al. (2016)	Monago and Akhidue (2002)	Eleazu et al. (2012)

– Not detected, * peeled seed, *dw* dry weight, *ww* wet weight

### Biological activities of kolaviron (KV)

A brief description of the biological activities of KV is given below; detail description (disease, dose, mode of administration, etc.) is given in Table 4.

**Table 4:** Biological activities of kolaviron

Disease/model	Animal/organ	Dose/mode of administration	Positive control	Major findings	References
*Protection against toxic agents*					
Liver					
Thioacetamide	Rats	100 mg/kg i.p	N/A	KV reduced the thiopental-induced sleep in thioacetamide-poisoned rats	Iwu et al. (1990a)
Carbon tetrachloride (CCl4)	Mice	100 mg/kg (N/A)	Vitamin E (100 mg/kg)	KV enhanced recovery from CCl4-induced hepatotoxicity by decreasing the extent of lipid peroxidation and also inducing the levels of phase II enzyme (e.g. cytosolic glutathione-*S*-transferase)	Adaramoye et al. (2008)
2-acetylaminofluorine (2-AAF)	Rats	100 mg/kg p.o	BHA (7.5 g/kg)	KV decreased the 2-AAF reduction of 5'-nucleotidase and glucose-6-phosphatase activities	Farombi et al. (2000)
Aflatoxin B1 (AFB1)	Rats	100, 200 mg/kg p.o	Vitamin C and E (100 mg/kg)	KV reduced the AFB1-induced malondialdehyde and lipid hydroperoxide formation	Farombi et al. (2005)
Sodium-arsenite	Rats	100, 200 mg/kg (N/A)	N/A	KV pre-treatment suppressed markers of oxidative stress and prevented the depletion of antioxidant defence system	Agboola et al. (2016)
Streptozotocin	Diabetic rats	100 mg/kg p.o	N/A	KV attenuated lipid peroxidation and apoptosis, increased the activity and levels of antioxidant defence system	Oyenihi et al. (2015)
Dimethyl nitrosamine (DMN)	Rats	100, 200 mg/kg p.o	curcumin (200 mg/kg)	KV lowered the DMN-induced activities of serum transaminases and γ-glutamyl tranferase	Farombi et al. (2009)
Diclofenac (DF)	Rats	100, 200, 400 mg/kg p.o	N/A	KV prevented or reduced the adverse effects of DF in the plasma, liver, and kidney of the rats	Alabi et al. (2017)
Sodium valproate (SVP)	Rats	200 mg/kg (N/A)	N/A	KV reduced the SVP-induced advanced oxidized protein products formation and restored the plasma surfydryl protein level. It also increased the activity and levels of antioxidant defence system	Ola and Adewole (2021)
Multiwalled carbon nanotubes	Rats	100 mg/kg	N/A	KV increased the antioxidant enzymes and enhance the glutathione levels and reduced malondialdehyde levels	Awogbindin et al. (2021)
Isoniazid, rifampicin, pyrazinamide and ethambutol	Rats	200 mg/kg	N/A	KV restored the antioxidant parameters (glutathione, glutathione peroxidase, glutathione-*S*-transferase and superoxide dismutase) and biochemical indices to near normal	Adaramoye et al. (2016)
Chloroquine (CQ)	Hepatocytes	% of tailDNA = 17.7, 53.0 µg/mL	vitamin C (1.7 µg/mL; quercetin (15.1 µg/mL)	KV decreased the CQ-induced strand breaks and base oxidation	Farombi (2006)
Kidney					
Carbon tetrachloride (CCl4)	Mice	100, 200 mg/kg i.p	vitamin E (100 mg/kg)	KV increased glutathione and superoxide dismutase levels	Adaramoye (2009)
Nevirapine	Rats	200 mg/kg p.o	vitamin C (250 mg/kg)	KV attenuated nephrotoxic effects and reduced activities of antioxidant enzymes (superoxide dismutase and catalase)	Offor et al. (2017)
Diclofenac	Rats	100, 200, 400 mg/kg p.o	N/A	KV reduced the toxic effect of DF on PGE_2_ release, plasma levels of creatinine, urea, glucose, and electrolytes, and attenuated renal tubular and oxidative damages	Alabi et al. (2018)
Gastrointestinal system					
Indomethacin and HCl/ethanol	Rats	100 mg/kg p.o	Ranitidine (50 mg/kg)	KV reduced the ulcers formation induced by indomethacin and HCl/ethanol and attenuated reduction of antioxidant defences	Olaleye and Farombi (2006)
Sodium arsenite	Rats	100, 200 mg/kg p.o	N/A	KV reduced the formation of malondialdehyde and activity of myeloperoxidase	Akinrinde et al. (2015)
Proton pump inhibition	Rats	200 mg/kg (N/A)	N/A	KV reduced the incidence of cold-restrained, aspirin. alcohol, and pyloric ligation-induced ulcers	Onasanwo et al. (2011)
Cardiovascular diseases					
Hypolipidemic effect in heart	Cholesterol-fed rats	100, 200 mg/kg p.o	Questran (100, 200 mg/kg)	KV exerted hypocholesterolaemic effect and reduced the relative weight of the heart, and decreased formation of malondialdehyde	Adaramoye et al. (2005)
Lower blood pressure	Hypertensive rats	200 mg/kg p.o	Lisinopril (2.3 mg/kg)	KV attenuated elevation in blood pressure and prevented dyslipidaemia	Uche and Osakpolo (2018)
	Rats	50, 100, 200 mg/kg (N/A)	Amlodipine (0.14 mg/kg)	KV reduced the elevated systolic, diastolic, and mean arterial pressures produced by ethanol and sucrose administration, decreased reduction of antioxidant defences, reduced cholesterol, triglycerides and LDL, and increased HDL	Olatoye et al. (2021)
Ischemia/reperfusion	Rats	200 mg/kg (N/A)	N/A	KV reduced the activity of antioxidant defences in ischaemic heart and levels of reactive species and malondialdehyde, increased Akt/protein kinase B, p-Akt/PKB (Ser 473), reduced p38 MAPK, caspase 3, and cleaved poly adenosine diphosphate ribose polymerase	(Oyagbemi et al. (2017)
	Isolated rat heart	15 min perfusion with 50 μg/mL KV	N/A	KV reduced p38 MAPK, total caspase 3, cleaved caspase 3 (Asp 175) and PARP cleavage, downregulated p-JNK1 (Tyr 185) and p-JNK 2 (Thr 183), and increased Akt/PKB and p-Akt/PKB (Ser 473)	Oyagbemi et al. (2018a)
Cardioprotective activity against toxic agents:					
Doxorubicin	Rats	100, 200 mg/kg p.o	N/A	KV reversed doxorubicin induced-increase in heart rate and prolonged QT, reduction of antioxidant status, increase of oxidative stress, inflammation and markers of cardiac damage	Oyagbemi et al. (2018c)
Cyclophosphamide (CP)	Rats	200, 400 mg/kg p.o	N/A	KV increased food consumption, body weight, and attenuated CP-induced biochemical and histological changes (higher cardiac troponin I, myeloperoxidase, malondialdehyde, hydrogen peroxide and lower antioxidant defences)	Omole et al. (2018)
Cobalt chloride	Rats	200 mg/kg p.o	Gallic acid (200 mg/kg)	KV prevented the toxic effects of CoCl_2_ by stimulating ERK expression and reversing Co-induced biochemical changes (kinase–myocardial band, lactate dehydrogenase, aspartate transaminase, xanthine oxidase, urea, creatinine, malondialdehyde, H_2_O_2_, nitric oxide, C-reactive protein expression, and reduced activities of antioxidant defences)	(Akinrinde et al. (2016)
Homocysteine	Rats	100, 200 mg/kg p.o	N/A	KV reversed homocysteine-induced reduction in heart rate, shortened QT and QTc intervals, and low voltage QRS	(Oyagbemi et al. (2016)
Amodiaquine, artesunate	Rats	100, 200 mg/kg p.o	N/A	KV did not prevent the cardiotoxicity and coronary risk effect caused by amodiaquine and artesunate	Ajani et al. (2008)
*Central nervous system*					
Protection against neurotoxins					
Atrazine (ATZ)	Human PC12 cells	35.3 µg/mL	N/A	KV demonstrates restoration in ATZ-induced alterations in the expression of apoptosis markers viz., p53, Bax, Bcl2, caspase-3, caspase-9, COX-2, c-Jun and c-fos	Abarikwu et al. (2011a)
	Human SH-SY5Y cells	35.3 µg/mL	N/A	KV prevented ATZ-induced increase in antioxidant defences and prevented ATZ-derived changes associated with apoptosis and in the expression of p53, Bax, Bcl-2, p21, and mRNA levels of caspase-3 and caspase-9	Abarikwu et al. (2011b)
Glucose	Isolated rat brain	0, 60, 120, 240 μg/mL	Metformin (N/A)	KV inhibited α-glucosidase and α-amylase activities, and intestinal glucose absorption, and attenuated oxidative-induced enzyme activities	Salau et al. (2021)
Methamphetamine	Rats	200 mg/kg p.o	N/A	KV delayed onset of methamphetamine-induced stereotypic movement and prevented destruction of pyramidal cells of the hippocampus	Ijomone et al. (2012)
Vanadium	Rats	100 mg/kg p.o	N/A	KV ameliorated vanadium-induced lipid peroxidation, reduced thiobarbituric acid–reactive substances, and increased activity of superoxide dismutase in all brain regions	Igado et al. (2012)
Phenytoin (PHT)	Rats	200 mg/kg p.o	Vitamin E (500 mg/kg)	KV reversed the PHT-mediated alterations in the haematology (haemoglobin, white blood cells, lymphocytes and mean corpuscular volume levels), brain antioxidant status (e.g. lipid peroxidation and hydrogen peroxide levels) and histomorphometry (reduced molecular layer and density of Purkinje cell)	Owoeye et al. (2014)
Sodium azide (NaN_3_)	Rats	200 mg/kg p.o	N/A	KV prevented NaN_3_-induced change in astroglia density and scar formation, alteration in glucose metabolism (glucose-6-phosphate dehydrogenase and lactate dehydrogenase levels) and antioxidant status	Olajide et al. (2015)
	Rats	200 mg/kg p.o	N/A	KV attenuated the NaN_3_-initiated destructive molecular cascades in prefrontal cortex via inhibition of stressor molecules and toxic proteins, prevention of stress related biochemical redox, preservation of neuronal integrity, cytoskeletal framework, and reduced the level of apoptotic regulatory proteins	Olajide et al. (2016)
Scopolamine	Rats	25, 50, 100 mg/kg p.o	Tacrine (5 mg/kg)	KV improved spatial learning in Morris water maze tests, ameliorated scopolamine-induced deficit in percentage alternation behaviour in the Y-maze test, and increase in lipid peroxidation, nitrite generation and decrease in glutathione and superoxide dismutase in the brain	Ishola et al. (2017)
Whisker removal-induced stress	Rats	200 mg/kg p.o	N/A	KV reversed Whisker removal-induced biochemical alterations (e.g. increased lipid peroxidation, reduced, catalase activities, glutathione levels), and histological abnormalities (cellular degeneration and necrosis) in the brain	Ibironke and Fasanmade (2016)
Maternal deprivation model	Rats	200 mg/kg p.o	N/A	KV attenuated negative effects caused by maternal deprivation (behavioural deficits, oxidative stress, degenerative changes, and astrocytosis in the prefrontal cortex and hippocampus)	Omotoso et al. (2020b)
Busulfan-induced episodic memory deficit model	Rats	200 mg/kg p.o	N/A	KV reversed busulfan-induced episodic cognitive deficit, decreased testicular/body weights and spermatogenesis. changes in androgenic hormones (testosterone, FSH, LH), dehydrogenase enzymes (3ß-HSD and 17ß-HSD), and normalized levels of reduced serotonin, dopamine, noradrenaline concentrations, elevated glutamate levels, acetylcholinesterase, monoamine oxidase-A and B activities	Oyovwi et al. (2021)
Rotenone-induced model of Parkinson’s disease	*Drosophila melanogaster*	100, 200, 300, 400, 500 mg/kg p.o	N/A	KV extended lifespan of flies, attenuated rotenone-induced inhibition of catalase, glutathione-*S*-transferase and acetylcholinesterase activities and depletion of total thiols content in flies, increase in H_2_O_2_ and nitric oxide levels and improved locomotor performance of flies	Farombi et al. (2018)
	Rats	200 mg/kg i.p	N/A	KV reversed the rotenone-associated locomotor impairment and exploratory deficits, motor/neuromuscular incompetence, striatal neurodegeneration, neurobiochemical imbalance, altered antioxidant defence system and neuroinflammation (regulation of COX-2 expression and interleukin and TNF-α levels)	Farombi et al. (2019)
	Rats	200 mg/kg p.o	N/A	KV suppressed the behavioural deficit and apomorphine-induced rotations associated with rotenone lesioning, attenuated the loss of nigrostriatal dopaminergic neurons and perturbations in the striatal glucose-regulated protein levels, and the redox imbalance in the gut and enhanced occludin immunoreactivity	Farombi et al. (2020a)
MPTP-induced Parkinson’s disease	Mice	200 mg/kg p.o	N/A	KV modulated striatal degeneration, behavioural impairment, antioxidant/redox imbalance and neuroinflammation implicated in the pathogenesis of PD via upregulation of DJ-1 secretion and inhibition of CD45R cells infiltration	(Farombi et al. (2020b)
Multiple sclerosis (cuprizone demyelination model)	Rats	200 mg/kg p.o	N/A	KV reversed cuprizone-induced reduction in the number of the line crossed, rearing frequency, rearing duration, centre square entry, and centre square duration, markers of oxidative stress, and histopathology changes	Omotoso et al. (2020a)
*Reproduction and infertility*					
Protection against reproductive toxins:					
Ethylene glycol monoethyl ether (EGEE)	Boar spermatozoa	IC_50_ = 29.4, 58.9 µg/mL	Vitamin C (176.1 µg/mL)	KV decreased H_2_O_2_ and malondialdehyde levels, improved spermatozoa characteristics and ameliorated oxidative damage in EGEE-treated spermatozoa	Adedara and Farombi (2014)
	Rats	100, 200 mg/kg p.o	Vitamin E (50 mg/kg)	Kolaviron exhibited protective effects against EGEE-induced reproductive toxicity by enhancement of antioxidant status and improvement in spermatozoa quantity and quality	Adedara and Farombi (2012, 2013)
Benzo-[*a*]-pyrene	Rats	100 and 200. mg/kg p.o	N/A	KV suppressed pro-inflammatory mediators and enhanced the antioxidant status, neuroendocrine function, sperm characteristics and improved the brain and testes architecture	Adedara et al. (2015)
Phenytoin	Rats	200 mg/kg (N/A)	Vitamin E (500 mg/kg)	KV restored antioxidant status and the functional indices of liver and testes to near control levels	Owoeye et al. (2015),
Nevirapine	Rats	200 mg/kg p.o	N/A	KV ameliorated the biochemical changes caused by nevirapine (sperm quality, elevation of serum aminotransferases and γ-glutamyl transferase activities, and decrease of antioxidant defences)	Adaramoye et al. (2013)
Butylphthalate (DBP)	Rats	200 mg/kg p.o	Curcumin (200 mg/kg)	KV provided protection against DBP-induced testicular oxidative damage (prevented the decline of antioxidant status, elevation of malondialdehyde, and decreased activity of glutamyl transferase), slowed down the DBP-induced decline of testosterone levels, and restored sperm functional indices	Farombi et al. (2007)
Cadmium	Rats	200 mg/kg p.o	Quercetin (10 mg/kg)	KV prevented Co-mediated decrease in sperm motility and epididymal sperm concentration and reversed the increased level of sperm abnormality to near control, reversed the Co-induced decrease in the body weight gain, testis and epididymis weights and its negative effect on antioxidant enzymes and markers of oxidative stress	Farombi et al. (2012),
Ethanol	Rats	200 mg/kg p.o	N/A	KV almost completely inhibited ethanol-derived testicular lipid peroxidation process and enhanced antioxidant status of the testis (i.e. reversed accumulation of malondialdehyde)	Adaramoye and Arisekola (2012)
Busulfan	Rats	50 mg/kg p.o	Rutin (30 mg/kg)	KV reversed busulfan-induced increase in oxidative stress, and preserved spermatogenesis and improved sperm quality	Abarikwu et al. (2021)
Multiwalled carbon nanotubes (MWCNT)	Rats	50, 100 mg/kg p.o	N/A	KV mitigated MWCNTs-induced inhibition of antioxidant enzyme activities increases in oxidative stress and inflammatory indices	Adedara et al. (2021)
Diabetes					
Hypoglycaemic effect	Diabetic rabbits	100 mg/kg i.p	Tolbutamide (500 mg/kg)	KV reduced fasting blood sugar levels in animals and inhibited rat lens aldose reductase activity in vitro	Iwu et al. (1990b)
	Diabetic rats	100 mg/kg p.o	N/A	KV decreased blood glucose levels, showed favourable effect on the plasma lipid profile, and decreased the streptozocin-induced increase in the activity of microsomal glucose-6-phosphatase and lipid peroxidation products	Adaramoye and Adeyemi (2006)
	Diabetic rats	100 mg/kg p.o	Glibenclamide (5 mg/kg)	KV reduced fasting blood glucose, α-amylase and HbA1c and attenuated the cardiac, renal and liver marker indices (increased serum creatine kinase, lactate dehydrogenase, creatinine, urea and alanine aminotransferase)	Adaramoye (2012)
Regeneration of pancreatic islets	Diabetic rats	100 mg/kg p.o	N/A	KV restored islet architecture in pancreatic β-cell through increase in the number of large and very large islets compared to diabetic control	Oyenihi et al. (2021)
Pain and inflammation					
Carrageenan-induced paw edema	Mice	50 mg/kg (N/A)	Indomethacin (10 mg/kg)	KV inhibited carrageenan-induced paw oedema at comparable activity to positive control	Tchimene et al. (2015a)
Pneumonia	BALB/c mice	400 mg/kg p.o	N/A	KV reversed the influenza-established nitrative stress (nitric oxide), the elicited cytokine storm (increased expression of pulmonary IL-1β, RANTES, IL-10, MCP-1, NF-κB, iNOS and COX-2) and restored the oxidized environment	Awogbindin et al. (2017)
	Rats	250, 500 mg/kg (N/A)	Ofloxacin (2.86 mg/kg)	KV showed anti-inflammatory effects with corresponding improvements in histopathological examinations, and antibacterial effect against *Klebsiella pneumonia*	Dozie-Nwakile et al. (2021)
Reduced symptoms of inflammation	Diabetic rats	100 mg/kg p.o	N/A	KV improved antioxidant status and abated inflammatory response by reducing the levels of proinflammatory cytokines and growth factor, lipid peroxidation product, and the restoring activities of erythrocyte antioxidant enzymes in the blood	Ayepola et al. (2014a)
	Sertoli cell lines	2.9, 5.9, 8.8, 14.7, 29.4, 58.9 µg/mL	N/A	KV modulated expressions of inflammatory marker genes (e.g. for various interleukins, and TNF-α, Tlr-4), inhibited transcription factors ERK1/2, p-JNK, NF-κB, and activated Akt expressions	Abarikwu (2014)
	U937 cells	25 µg/mL	N/A	KV reduced the production of H_2_O_2_ and H_2_O_2_-induced secretion of nitric oxide, TNF-α, IL-1 and IL-6, and improved overall viability of U937 cells	Okoko (2018)
Anaesthesia	Guinea pig	0.33, 0.66,1.00 mg/kg i.d	Xylocaine (0.33, 0.66,1.00 mg/kg)	KV induced local anaesthesia at comparable levels to xylocaine	Tchimene et al. (2015b)
Immunomodulatory activity					
Leukopenia	Rats	250, 500 mg/kg p.o	Levamisol (25 mg/kg)	KV inhibited delayed-type hypersensitivity, increased the primary and secondary sheep erythrocytes-specific antibody titres, ameliorated the cyclophosphamide-induced leukopenia and increased the proportion of lymphocytes count, and increased the rate of excision wound closure and reduced epithelialization period	Nworu et al. (2008)
Delay of influenza symptoms	BALB/c mice	400 mg/kg, p.o	N/A	KV increased weight and prolonged life expectancy of infected mice, improved lung aeration and reduced lung consolidation, inflammatory cells infiltration, and attenuated myeloperoxidase activity and nitric oxide production	Awogbindin et al. (2015)
Cancer					
Benign prostate hyperplasia	Mice	100, 200 mg/kg p.o	Finasteride (0.07 mg/kg)	KV had decreased prostate weights compared with the normal control and ameliorated most of the disease parameters, including serum levels of prostate specific antigen, estradiol, testosterone, testosterone/estradiol ratio, and prostatic levels of total proteins	Kalu et al. (2016)
Antiparasitic activity					
Infection by *Plasmodium bergheii*	Mice	100, 200 mg/kg p.o	Chloroquine (10 mg/kg)	KV suppressed *P. berghei*-infection, increased the mean survival time of the infected mice, ameliorated anaemia, oxidative stress (e.g. elevated malondialdehyde), and infection-related decrease in antioxidant status	Oluwatosin et al. (2014)
	Mice	25, 50, 100, 200 mg/kg p.o	Artemisinin (15 mg/kg)	KV increased the average life span of the infected mice and supressed the *P. berghei* infection	Konziase (2015)
* Plasmodium falciparum*	In vitro assay	IC_50_ = 0.1–88.3 µg/mL	Quinine (0.04–0.09 µg/mL)	KV displayed potent inhibitory activity in vitro against *P. falciparum* proliferation, and low cytotoxicity against KB 3–1 cell line	Konziase (2015)
Infection by *Trypanosoma congolense*	Rats	200 mg/kg p.o	Chrysin (40 mg/kg)	KV showed inhibition of *T. congolense* in both in vivo and in vitro conditions, and it downregulated the expression trypanothione reductase gene	Timothy et al. (2021)

*p.o.* perorally, *i.p.* intraperitoneally, *i.d.* intradermally, *N/A* information not available

#### Hepato-, nephro-, and gastrointestinal-protective activity

Hepatoprotective effect is one of the major area where KV was tested. The biflavonoid was investigated in animal models to protect the liver from a broad spectrum of hepatotoxic agents. Despite the intensive research, the exact mode of hepatoprotective action of KV is still not fully understood. Some authors proposed direct antioxidant mechanism (e.g. via KV’s ability to scavenge free radicals) (Alabi and Akomolafe 2020), while others pointed out that KV enhances activity of drug-detoxifying enzymes (KV increases the activity of UDP-glucuronosyl transferase and glutathione *S*-transferase) (Olatunde Farombi 2000). Farombi et al. (2009) also suggested that its effect may be achieved through inhibition of cyclooxygenase (COX) and inducible nitric oxide synthase (iNOS) expression. Similarly, KV was also tested in the scenario of renal (Adaramoye 2009; Adedara et al. 2015; Offor et al. 2017; Alabi et al. 2018) and gastro-intestinal (Olaleye and Farombi 2006; Onasanwo et al. 2011; Akinrinde et al. 2015) protection in animal models against similar toxic agents as in the case of liver toxicity tests. Both nephroprotective and gastro-protective effect is presumably exerted via similar mode of action. Apart from mechanisms discussed above, it was also suggested that KV interferes with regulation of such structures as C-reactive proteins (CRP) and extracellular signal regulated kinase (ERK) (Ayepola et al. 2014b; Akinrinde et al. 2016; Oyagbemi et al. 2018b). In the case of gastrointestinal protective activity, KV was suggested to inhibit proton pump, thus providing anti-ulcerogenic effect.

#### Effect on heart and cardiovascular disorders

In early studies, KV was shown to produce hypolipidaemic effect and to reduce the relative heart weight of cholesterol-fed rats. Its activity was comparable to that of cholestyramine (questran), a commonly used hypocholesterolemic drug (Adaramoye et al. 2005). Additionally, KV was found to lower blood pressure in hypertensive rats (Uche and Osakpolo 2018; Olatoye et al. 2021). In other studies dealing with animal ischemic/reperfusion model, KV demonstrated to attenuate the heart injury through interference with apoptotic pathway (e.g. caspase reduction/cleavage), and reperfusion injury signaling kinase (RISK) (Oyagbemi et al. 2017, 2018a). In a more recent study, KV also reduced cardiovascular injury in fructose-streptozotocin induced diabetic rats (Adoga et al. 2021). Furthermore, KV showed cardioprotective effect in animal models against various cardiotoxic agents, including antitumour drugs, and antimalarial agents (e.g. amodiaquine and artesunate) (Ajani et al. 2008).

#### Effects on central nervous system (CNS)

The early studies of KV were focused on in vitro determination of its protective activity against atrazine in certain neurological cell cultures (e.g. human dopaminergic SH-SY5Y and PC12 cells) (Abarikwu et al. 2011a, b). The CNS experiments were afterwards transferred to animal models, where KV showed neuroprotective effect against several neurotoxins. It was suggested that antioxidant effect (i.e. enhancement of antioxidant defences) might be the major mechanism of its beneficial action, though other modes were proposed as well (such as inhibition of stressor molecules and toxic proteins production). KV also demonstrated positive results in the animal models of cuprizone-induced multiple sclerosis. Again, its beneficial effect was explained by antioxidant-related action (Omotoso et al. 2018a, b, 2019). A neuroprotective effect was also observed in various rat models of CNS disorders. It was suggested, that KV might exert its neuroprotective effect through anti-inflammatory and antiapoptotic mechanisms. Additionally, KV was also suggested to be a potential inhibitor of acetylcholinesterase (AChE) (Ijomone and Obi 2013; Akinmoladun et al. 2018), though this was deduced only on the basis of reduced staining activity of AChE and not by enzyme-binding study. Moreover, very recently KV indicated an anti-amyloid activity via destabilization of the assembled Aβ particles in a molecular docking study (Adewole et al. 2021a).

#### Effect on reproduction and infertility

*Garcinia kola* is relatively widely used in traditional medicine as an aphrodisiac. Corresponding with this fact, studies have been focused on examining the effect of present substances on reproductive properties. KV was found to prevent testicular damage and decline of sex hormones upon administration of various toxic agents. Administration of these agents resulted in increased levels of antioxidant/detoxifying enzymes (catalase, superoxide dismutase, glutathione S-transferase) and markers of oxidation (e.g. elevated hydrogen peroxide and malondialdehyde). Additionally, the rats that had been treated with KV also showed improved semen characteristics (e.g. sperm count). It was also found out, that KV has lowered the negative effect of EGEE on activities of 3β-hydroxysteroid dehydrogenase (3β-HSD) and 17β-hydroxysteroid dehydrogenase (17β-HSD), enzymes that are associated with production of steroidal hormones (e.g. testosterone) (Adedara and Farombi 2013).

#### Diabetes

Investigations were made to figure out if KV can act as a potential source of diabetes treatment. The compound showed a hypoglycaemic effect in alloxan-induced diabetic rabbits and streptozotocin-induced diabetic rats (Iwu et al. 1990b; Adaramoye and Adeyemi 2006; Adaramoye 2012). Though there is no generally accepted mechanism of action yet, KV was suggested to produce its antidiabetic effect via inhibition of α-glucosidase and α-amylase activities (Iwu et al. 1990b; Salau et al. 2020). Recent study has also suggested that KV may play a regenerative role in pancreatic islets in streptozotocin-induced diabetic rats (Oyenihi et al. 2021). Other studies focused on the KV ability to reduce secondary pathology complications associated with diabetes, including hepatoxicity, nephrotoxicity, and cardiotoxicity. These activities have been discussed in previous sections.

#### Pain and inflammation

Anti-inflammatory activity of KV was firstly studied in an carrageenan-induced paw oedema mice model (Olaleye et al. 2010; Tchimene et al. 2015a). According to the subsequent tests on cell lines, it was suggested that KV might be the most active anti-inflammatory principle of *G. kola*, interfering with the normal production of pro-inflammatory mediators such as prostaglandins (via COX enzymes inhibition), nitric oxide, interleukins, tumour necrosis factor α (TNF-α), monocyte chemotactic protein-1 (MCP-1), and vascular endothelial growth factor (VEGF) (Olaleye et al. 2010; Abarikwu 2014; Ayepola et al. 2014b, a; Awogbindin et al. 2017; Okoko 2018). Recent animal study revealed that KV have decreased inflammation in pneumonia-like *Klebsiella* infection induced in wistar rats (Dozie-Nwakile et al. 2021). Additionally, KV was found to reduce neuroinflammation in BV2 microglia/HT22 hippocampal neuron co-culture, exerting its activity via the same mechanisms mentioned above. Also, KV showed to possess a certain analgesic effect (Tchimene et al. 2015b). It was later discovered that its pain-relieving activity may not probably be associated with COX-2 inhibition, but rather involves nitrergic and ATP-K^+^ sensitive pathways (Ibironke and Fasanmade 2015).

#### Immunomodulatory activity

There are a few studies addressing the immunomodulatory activity of KV. In the first report, research on immunocompetent and immunocompromised models in rats was carried out, where KV showed inhibition of delayed-type hypersensitivity and increase in the primary and secondary sheep erythrocytes-specific antibody titers. The results showed that administration of KV ameliorated the cyclophosphamide-induced leukopenia and increased the number of white blood cells (Nworu et al. 2008). In other studies, KV delayed the development of the clinical symptoms of influenza in the infected mice (Awogbindin et al. 2015). Apart from other mechanisms discussed above (e.g. inhibition of COX-2, interleukins, and cytokines production), it was suggested that KV is capable of fostering the CD4^+^ response (Awogbindin et al. 2017).

#### Cancer

Only a few studies regarding KV anticancer activity exist, though vast majority of them aim specifically at determining effect on biochemical parameters of benign prostatic hyperplasia in rats. KV showed a similar effect on serum levels of prostate specific antigen, total prostatic proteins, prolactin, oestradiol, testosterone, testosterone/oestradiol ratio, urea, and creatinine as the control finasteride (Kalu et al. 2016; Winner et al. 2016). Since antiandrogen finasteride is an 5α-reductase inhibitor, it was suggested that KV has the same mechanism of action. Yet, the therapeutic efficacy of KV in benign prostate hyperplasia (BPH) are far from conclusive. On top of that, it is worth to note that if indeed KV was an 5α-reductase inhibitor it would contradict the traditional aphrodisiac ethnomedicinal indication of *G. kola*. Recently, KV was also found to protect U937 cell and macrophages from bromate-induced cytotoxicity in an in vitro study (Okoko and Ndoni 2021). Moreover, histone deacetylase inhibitory activity was shown in an in silico model (Adewole et al. 2021b).

#### Antiparasitic activity

Although *G. kola* is commonly used in folk medicine to treat malaria, there are relatively few studies on its antimalarial effect. KV showed anti-malarial activities by suppressing *Plasmodium bergheii* in infected laboratory mice (Oluwatosin et al. 2014; Tshibangu et al. 2016). Of all KV components, GB1 exhibited the almost the same in vitro antimalarial effectivity on *P. falciparum* as quinine. In the in vivo test, it was observed that GB1 significantly increased the average life span of *Plasmodium*-infected mice (Konziase 2015). Recently, KV was also showed to be effective against *Trypanosoma* infections (e.g. *T. congolense*) both in vitro and in vivo. It has been suggested that KV may exert its antitrypanosomal activity by interfering with trypanothione reductase, an enzyme responsible for homeostasis maintenance (Timothy et al. 2021).

#### Anti-snake venom activity

Anti-snake venom activity forms a relatively narrow area of KV research. As far as we know, only one study addressed this issue. Quite recently Okafor and Onyike (2020) suggested that the KV may produce inhibitory effect against hydrolytic enzymes of *Naja nigricollis* venom, namely phospholipase A2 (PLA2), protease, hyaluronidase and l-amino acid oxidase, and thus also neutralize their myotoxic, oedemic, haemolytic and procoagulant effects. However, KV was assayed at quite high doses (venom:KV 1:5 w/w) and reasonable inhibition was only observed in the case of PLA2. It is questionable whether these high doses of KV are clinically relevant.

### Biological activities of amentoflavone

Amentoflavone is a widely studied biflavonoid. It is quite abundant in nature across various plant families, including—Ginkgoaceae, Selaginellaceae, Cupressaceae, Euphorbiaceae, Podocarpaceae, and Calophyllaceae (Yu et al. 2017). The main area where amentoflavone has been studied is anti-inflammatory, antitumour, antidiabetic, antifungal, antiviral, and neuro- and cardio-protective activities. Amentoflavone was found to interfere with levels of inflammatory mediators (e.g. nitric oxide, malondialdehyde, reduced glutathione, tumour necrosis factor alpha (TNF-α), and prostaglandin E-2) in various lipopolysaccharide-stimulated cell lines (Ishola et al. 2013). Additionally, amentoflavone was also reported to inhibit the production of proinflammatory interleukins, including IL-1β and IL-6 (Abdallah et al. 2015). As of yet, precise mode of its anti-inflammatory action has not been established. Amentoflavone have been tested for cytotoxic effect against various cancer cell lines. Several mechanisms of its anticancer action have been proposed, including induction of cell cycle arrest, apoptosis (e.g. interference with caspase-3), inhibition of fatty acid synthase (FASN) and phosphorylation of protein kinase B (PKB), and downregulation of HER2 protein (Lee et al. 2013). Amentoflavone was also suggested to regulate glucose level, production of insulin and to possess pancreas-regenerating properties in diabetic mice (Su et al. 2019). It was indicated that it may exert its antidiabetic effect by inhibiting protein tyrosine phosphatase 1 (PTP1B) (Na et al. 2007). Amentoflavone demonstrated neuroprotective effect in various experiments. This activity may be related to interference with the receptors for serotonin, adrenaline, and GABA. Amentoflavone also showed protective activity against cardiovascular dysfunction in high fructose and fat diet induced metabolic syndrome rats. Administration decreased systolic blood pressure, left ventricular internal diameter and posterior wall thickness in diastole, increased fractional shortening and decreased ejection fraction, relative wall thickness, estimated left ventricular mass, cardiac stiffness and wet weight (Qin et al. 2018). Amentoflavone was also shown to reduce lipid accumulation and oxidized low density lipoprotein (ox-LDL) uptake in HUASMCs and THP-1 cells. It was suggested that amentoflavone acts as an inhibitor of proliferator-activated receptor gamma (PPARγ) protein/cluster of differentiation 36 (CD36) signaling pathway (Zhuang et al. 2021). Additionally, amentoflavone was found out to inhibit phosphodiesterase in rat adipose tissue (Saponara and Bosisio 1998). Amentoflavone also showed antimicrobial activity against various fungal pathogens, including *Candida albicans*, *Saccharomyces cerevisiae*, and *Trichosporon beigelii* (Hyun et al. 2006). Furthermore, it demonstrated antiviral effect, e.g. against, coxsackievirus B3 (Wilsky et al. 2012), dengue virus (Coulerie et al. 2013), HIV (Lin et al. 1997), and SARS-CoV 3CL^pro^ (Ryu et al. 2010). As with the other biflavonoids mentioned in this review, amentoflavone, although possibly showing promising results in many of the in vitro and in vivo tests, has not yet been subjected to clinical trials and therefore its therapeutic efficacy is far from conclusive.

### Biological activities of volkensiflavone/morelloflavone

It seems the biflavonoids volkensiflavone and morelloflavone display similar pharmacological properties as their related structure KV. However, the extent of research on them is far more limited. Their biological activities are summarized in Table 5. Perhaps the most widely studied area of these biflavonoids is antibacterial activity, though in the available in vitro studies, they are showing rather low efficiency. Both showed an ability to lower the minimal inhibitory concentration of norfloxacin against *Staphylococcus aureus* (E Silva et al. 2021). Anti-bacterial activity (e.g. against *S. aureus*, but also *Bacillus subtilis*, *Pseudomonas aeruginosa* and *Escherichia coli*) of volkensiflavone and morelloflavone and their glycosylated versions was also reported elsewhere (Trisuwan et al. 2013; Jamila et al. 2014). Interestingly, some of the glycosylated analogues of volkensiflavone and morelloflavone (e.g. (2*R*,3*S*)-volkensiflavone-7-*O*-β-acetylglucopyranoside and (2*S*,3*S*)-morelloflavone-7-*O*-β-acetylglucopyranoside) did not demonstrate any notable antibacterial activity (Mountessou et al. 2018). Volkensiflavone demonstrated antiplasmodial activity in comparison to cholorquine. Morelloflavone showed approx. three- to ten-fold weaker activity than volkensiflavone (Azebaze et al. 2015). Contrastingly, Bezerra et al. (2021) reported morelloflavone to have activity against *Leishmania amazonensis*. Contrasting results were also found in another study, where volkensiflavone showed superior activity over morelloflavone against *Leishmania infantum*, but also other parasites (i.e. *Trypanosoma brucei brucei* and *T. cruzi*), while morelloflavone was not active at all (Mbwambo et al. 2006). Volkensiflavone and morelloflavone were also found to produce a vasodilatation via relaxation on aorta rings in a rat model. Both compounds were also suggested to be of interest as potential treatment for increased blood pressure and erectile disfunction (Brusotti et al. 2016). Moreover, their in vitro and in vivo atheroprotective effect caused by regulated interaction between oxidized low density lipoprotein (LDL) molecule and macrophages has been described as well (Tabares-Guevara et al. 2017). Volkensiflavone and morelloflavone were also tested in few in vitro and in vivo antitumour assays. Morelloflavone inhibited microvessel sprouting of endothelial cells in the mouse aortic ring assay and formation of new blood microvessels induced by VEGF in the mouse Matrigel plug assay. It also inhibited tumour growth and tumour angiogenesis of prostate cancer cells (PC-3) in xenograft mouse tumour model (Pang et al. 2009). Additionally, morelloflavone and its glycosylated variants demonstrated moderate antitumour effect in C6 cells (Li et al. 2016). Both volkensiflavone and morelloflavone displayed cytotoxicity in the SW-480 colon cancer cell line (Baggett et al. 2005). More recently, a biflavonoid fraction from *Garcinia madruno*, that was composed of morelloflavone (65%), volkensiflavone (12%), GB 2a (11%), fukugiside (6%) and amentoflavone (0.4%) demonstrated neuroprotective activity in a transgenic mouse model of Alzheimer’s disease (e.g. reduced deposition of Aβ particles, β-secretase-mediated cleavage of amyloid precursor protein, tau pathology, astrogliosis and microgliosis). Additionally, the mice administered with the biflavonoid mixture showed better behavioural patterns in comparison to the control group (Sabogal-Guáqueta et al. 2018).

**Table 5:** Biological activities of volkensiflavone/morelloflavone

Disease/model	Animal/organ	Efficacious dose/mode of administration		Positive control	Major findings	References
		Volkensiflavone	Morelloflavone			
In vitro antibacterial activity	*Staphylococcus aureus*	MIC = 135.1	MIC = 34.8	Vancomycin (724 µg/mL), streptomycin (290.8 µg/mL), gentamycin (3.6–15 µg/mL)	Volkensiflavone and morellovlavone and their glycosylated derivatives showed in vitro antimicrobial activity against the corresponding bacteria	Jamila et al. (2014)
	*Bacillus subtilis*	MIC = 135.1	MIC = 34.8			
	*Pseudomonas aeruginosa*	MIC = 33.8	MIC = 139.1			
	*Escherichia coli*	MIC = 135.1	MIC = 34.8			
In vitro antiparasitic activity	*Plasmodium falciparum*	IC_50_ = 25.9 μg/mL	IC_50_ > 35.6 μg/mL	N/A	Volkensiflavone and morellovlavone showed in vitro antimalarial and antitrypanosomal activity	Mbwambo et al. (2006)
	*Leishmania infantum*	IC_50_ > 34.6 μg/mL	IC_50_ > 35.6 μg/mL	N/A		
	*Trypanosoma brucei*	IC_50_ = 20 μg/mL	IC_50_ > 35.6 μg/mL	N/A		
	*Trypanosoma cruzi*	IC_50_ = 30.3 μg/mL	IC_50_ > 35.6 μg/mL	N/A		
In vitro antiviral activity	HIV	No activity	3.8 μg/mL		Morelloflavone (but not volkensiflavone) demonstrated in vitro anti-HIV-1 activity	Lin et al. (1997)
*Cardiovascular disorders*						
Vasodilatation	Rats	150 mg/kg (N/A)	150 mg/kg (N/A)	Viagra® (5 mg/kg)	Morelloflavone induced a relaxation on aorta rings	Brusotti et al. (2016)
Atheroprotection	Vascular smooth muscle cell migration (VSCM) test	N/A	0, 0.6, 6, 60 μg/mL	N/A	Morelloflavone blocked injury-induced neointimal hyperplasia via the inhibition of VSMC migration, without inducing apoptosis or cell cycle arrest	Pinkaew et al. (2009)
	LDL receptor-lacking mice	N/A	4 mg/kg p.o	N/A	Morelloflavone reduced the atherosclerotic areas of the mouse aortas without changing plasma lipid profiles or weights. It also reduced the number of vascular smooth muscle cells in the atherosclerotic lesion	(Pinkaew et al. (2012)
	Mice	70 mg/kg i.p.^a^		N/A	Biflavonoid mixture provided atheroprotective effects by positively affecting atheroligand formation, atheroreceptor expression, foam cell transformation, and prooxidant/proinflammatory macrophage response	Tabares-Guevara et al. (2017)
Hypercholesterolemia	Rabbits	N/A	7, 13, 26 mg/kg p.o	N/A	Morelloflavone reduced the increase in plasma cholesterol, triglycerides and thiobarbituric acid-reactive substances, and reversed the intimal thickening within aortas	Decha-Dier et al. (2008)
*Inflammation and pain*						
Analgesic activity	Formalin test in mice	10 mg/kg i.p. (% inhibition = 87.7)	10 mg/kg i.p. (% inhibition = 34.2)	Indomethacin (10 mg/kg i.p.; % inhibition = 74.4)	Volkensiflavone and morelloflavone exhibited analgesic activity in relation to the second phase (inflammatory pain) of the formalin test	Luzzi et al. (1997)
*Cancer*						
Antitumour activity	Mice PC-3 xenograft tumours	N/A	8 mg/kg (N/A)	N/A	Morelloflavone inhibited microvessel sprouting of endothelial cells in the mouse aortic ring assay and formation of new blood microvessels induced by VEGF in the mouse Matrigel plug assay. It also inhibited tumour growth and tumour angiogenesis in the xenograft model	Pang et al. (2009)
	Rat glioma C6 xenograft tumours	N/A	800 μg/kg in combination with cisplatin (1 mg/kg)	N/A	Morelloflavone and its glycosylated variants demonstrated antitumour effect in C6 cells	Li et al. (2016)
	SW-480 colon cancer cell line	IC_50_ = 100 µg/mL	IC_50_ = 49.5 µg/mL	N/A	Volkensiflavone and morelloflavone displayed cytotoxicity in the SW-480 colon cancer cell line	Baggett et al. (2005)
*Central nervous system*						
Alzheimer’s disease	Mice	25 mg/kg i.p.^b^		N/A	Biflavonoid mixture reduced Aβ paricles deposition, BACE1-mediated cleavage of APP (CTFβ), tau pathology, astrogliosis and microgliosis in different regions of the brain, and reduced emotional disorders	Sabogal-Guáqueta et al. (2018)

*p.o.* perorally, *i.p.* intraperitoneally, *N/A* information not available

^a^Tested as a mixture of morelloflavone (85%), volkensiflavone (10%), and amentoflavone (5%)

^b^Tested as a mixture of morelloflavone (65%), volkensiflavone (12%), GB 2a (11%), fukugiside (6%), and amentoflavone (0.4%)

### Biological activities of garcinol

#### Cancer

Garcinol is attracting a scientific interest mainly due to its ability to inhibit histone acetyltransferase (HAT), a novel drug target in cancer research. As a HAT inhibitor, garcinol was found effective at hindering the process of non-homologous end joining in the DNA repair mechanism, ultimately causing apoptosis of the cancer cells (Oike et al. 2012; Schobert and Biersack 2019). Other suggested mechanisms of garcinol’s anticancer effect is interference with NF-κB, iNOS, COX-2 (especially in the inflammatory-induced cancers, such as colorectal cancer), VEGF, and signal transducer and activator of transcription 3 (STAT-3) pathway (Liu et al. 2015; Schobert and Biersack 2019). In vivo and in vitro anti-cancer properties of garcinol have been quite recently and exhaustively reviewed by Aggarwal et al. (2020) and Schobert and Biersack (2019). Therefore, only the most important studies and those published after 2019 are summarized in Table 6.

**Table 6:** Biological activities of garcinol

Disease/model	Animal/organ	Efficacious dose/mode of administration	Positive control	Major findings	References
*Cancer*					
Lung	H441 and A549; non-small cell lung cell cancer (NSCLC) lines	3.01; 6.03; 12.06 µg/mL	N/A	Garcinol diminished the ability of the NSCLC cell lines to form spheres and colonies, and reduced their viability	Huang et al. (2018)
	Xenograft mouse model (NOD/SCID mice + H441 tumour spheres	5 mg/kg i.p	N/A	Garcinol inhibited tumour growth in the xenograft model	
Leukaemia	HL-60 cells	IC_50_ = 5.7 µg/mL	Curcumin IC_50_ = 7.2 µg/mL	Garcinol displayed in vitro growth inhibitory effects against human leukaemia HL-60 cells	Pan et al. (2001)
Pancreas	Transgenic pancreatic cancer (PC) mouse	0.05% in diet	Gemcitabine 100 mg/kg, i.p Garcinol 0.05% in diet + gemcitabine 100 mg/kg i.p	Mice administered with garcinol showed reduction in tumour volumes and reduced number of advanced pancreatic neoplasia. It also retarded the progression of PC, arresting the cancer in the earlier stages, improving prognosis and survival	Saadat et al. (2018)
Colon	HT-29 cells—viability Scratch test	IC_50 (24 h)_ = 24.7 µg/mL IC_50 (48 h)_ = 16.3 µg/mL (12.06; 24.11; 36.2 µg/mL)	N/A	Garcinol elevated apoptosis and inhibited HT-29 cells proliferation, angiogenesis, and invasion by suppressing the mPGES-1/PGE2/HIF-1α signalling pathways	Ranjbarnejad et al. (2017)
Azoxymethane dextran sodium sulphate induced colon cancer	C57BL/6 J mice	High fat diet (HFD) + garcinol 0.05% in diet normal diet (ND) + garcinol 0.05%	Normal diet high-fat-diet	Garcinol ameliorated obesity-promoted colon carcinogenesis	Lee et al. (2021)
Prostate	DU-145, PC-3, LNCaP	1.5; 3.04; 6.03; 12.06 µg/mL	N/A	Garcinol induced apoptosis and inhibited autophagy in human prostate cancer cells	Wang et al. (2015)
	Xenograft mouse model (nude mice + PC-3 cells)	50 mg/kg/d o.g. or i.p	N/A	Garcinol reduced the tumour size via inhibiting autophagy and inducing apoptosis	
Breast	Xenograft mouse model Balb/c mice + 4T1 cells	Synergy: Taxol® 5 mg/kg i.p. + garcinol 1 mg/kg i.g	Taxol® 5 mg/kg i.p.)	Garcinol enhanced Taxol®-stimulated G2/M phase arrest and the inhibition of caspase-3/cytosolic Ca^2+^-independent phospholipase A2 and NF-κB/Twist-1 drive downstream events including tumour cell repopulation, survival, inflammation, angiogenesis, invasion, and epithelial–mesenchymal transition	Tu et al. (2017)
Cervix	Hela;SiHa cells	IC_50_ = 32.5; 31.2 µg/mL	N/A	Garcinol suppressed cell viability, colony formation, invasion, migration, cell cycle progression, and promoted cell apoptosis in vitro	Zhao et al. (2018)
	Xenograft mouse model BALB/c nu/nu mice + Hela cells	1; 2 mg/kg i.p	N/A	Garcinol inhibited tumour growth in xenograft model	
Head and neck carcinoma (HNSCC)	CAL27 cells	6.03; 15.07; 30.14 µg/mL	N/A	Garcinol inhibited various inflammatory mediators involved in HNSCC progression (e.g. inhibition of STAT3 and NF-κB activation) and reduced cell viability and induced apoptosis in HNSCC cells	Li et al. (2013)
	Xenograft mouse model (athymic nu/nu male mice + CAL27 cells)	1;2 mg/kg i.p	N/A	Garcinol inhibited the growth of human HNSCC xenograft tumours	
Skin 7, 12-dimethylbenz[*α*]anthracene (DMBA)/TPA induced	Mice	1.2, 3 mg topically	N/A	Garcinol reduced TPA-induced expression of iNOS and COX-2, and nuclear translocation of NF-κB and its subsequent DNA binding. It also attenuated DMBA/TPA-induced mouse skin tumour promotion	Hung et al. (2015)
*Anti-inflammatory activity*					
Skin inflammation *12-O* tetradecanoylphorbol-13-acetate (*TPA*) induced ear oedema	Mouse	1.2, 3 mg topically	N/A	Garcinol reduced TPA-induced expression of iNOS and COX-2, and nuclear translocation of NF-κB and its subsequent DNA binding. It also attenuated DMBA/TPA-induced mouse skin tumour promotion	Hung et al. (2015)
Lipopolysaccharide (LPS) induced inflammation	BALB/c mice	10 mg/kg i.p	N/A	Garcinol enhanced LPS-induced expression of TNF-α and IL-6, exacerbated LPS-induced lung injury, increased LPS-induced elevation of plasma alanine aminotransferase and blood urea nitrogen, and reduced the survival rate of LPS-challenged mice	Wang et al. (2016)
Liver inflammation	C57BL/6N mice	20 mg/kg/day i.p	N/A	Garcinol decreased liver damage and improved survival in mice with acute liver failure	Ferriero et al. (2018)
Obesity-related inflammation	C57BL/6 mice	0.1 and 0.5% garcinol in high fat diet	N/A	Garcinol reversed high-fat diet-induced gut dysbiosis and controlled inflammation by increasing the intestinal commensal bacteria (e.g. *Akkermansia* spp.)	Lee et al. (2019)
*Neurodegenerative disorders*					
Parkinson disease (PD)					
6-Hydroxydopamine (6-OHDA) induced	C57BL/6 J mice	2; 5 mg/kg p.o	2 mg/kg, i.p., anacardic acid; 100 mg/kg p.o., curcumin	Garcinol reduced the L-DOPA-induced dyskinesia in affected mice	Ryu et al. (2018)
1-Methyl-4-phenyl-1,2,3,6-tetrahydropyridine (MPTP) -induced	Mice	10; 25 mg/kg i.p	N/A	Garcinol blocked the parkinsonian motor behavioural deficits (including akinesia, catalepsy, and rearing anomalies), and prevented degeneration of dopaminergic cell bodies and reduced inflammatory markers in the substantia nigra	Chetia Phukan et al. (2022)
Epilepsy					
Pentylenetetrazole (PTZ) induced	C57BL/6 mice	50, 100 or 200 mg/kg i.p	150 mg/kg, i.p. valproate	Garcinol reduced seizure scores and mortality rates, downregulated apoptotic proteins and caspase-3, enhanced GABA_A_ and GAD65 while it suppressed BDNF and TrkB, and enhanced the performance of mice in Morris water maze tests	Hao et al. (2016)
Neuropathic pain					
Lumbar fifth spinal nerve ligation (SNL) induced	Sprague–Dawley rats	100 μg/kg intrathecally	N/A	Garcinol inhibited the SNL-induced microglia activation in the spinal cord and ameliorated the neuropathic pain, attenuated the neuroinflammation (expression of interleukin interleukins, iNOS)/NO, and COX-2/PGE_2_), and inhibited lipopolysaccharide-stimulated inflammatory response in microglia in *vitro*	Wang et al. (2017)
In vitro	Primary rat microglial cells	3.04 mg/mL	N/A		
*Drug withdrawal*					
Reconsolidation of a cocaine-associated memory	Sprague–Dawley rats	10 mg/kg i.p	N/A	Garcinol impaired the reconsolidation of the cocaine-associated cue memory. It had no effect on drug-induced cocaine-seeking, but was capable of blocking the initial conditioned reinforcing properties of the cue and prevented acquisition of new response	Monsey et al. (2017)
*In vitro antiviral activity*					
	HIV—p300; PCAF transcriptional coactivators	IC_50_ = 4.5 µg/mL	N/A	Garcinol inhibited histone acetylation of HIV infected cells, and consequently inhibited the multiplication of HIV	Mantelingu et al. (2007)
	HIV‐1 reverse transcriptase	IC_50_ = 5.2 µg/mL	RDS1759 IC_50_ = 3.1 µg/mL (8.7 μM)	Garcinol showed inhibition of HIV-1 reverse transcriptase-associated ribonuclease H enzyme	Corona et al. (2021)
	Influenza A virus	30.14 µg/mL	Ribavirin—(12.2 µg/mL (50 μM), anacardic acid—17.1 µg/mL (50 μM)	Garcinol showed anti-influenza effect by blocking the interaction of PB2 with acetyl-CoA	Hatakeyama et al. (2014)
*In vitro antimicrobial activity*					
	*S. aureus* MRSA; MSSA clinical isolates	MIC = 6.25–25 μg/mL	Vancomycin MIC = 0.8–6.25 μg/mLgentamicin 1.57—> 25 μg/mL	Garcinol showed in vitro antimicrobial activity against the corresponding bacteria	Iinuma et al. (1996)
	*E. coli*	MIC = 25 μg/mL	MIC > 25 μg/mL, vancomycin; MIC = 25 μg/mL, gentamicin		
	*Bacillus cereus*	MIC = 1.5 μg/mL	N/A	Garcinol showed in vitro antimicrobial activity against the corresponding bacteria and antioxidant action in DPPH (1,1-diphenyl-2-picrylhydrazyl) radical assay	Negi and Jayaprakasha (2004)
	*B. coagulans*	MIC = 2 μg/mL	N/A		
	*B. subtilis*	MIC = 2 μg/mL	N/A		
	*S. aureus*	MIC = 1.5 μg/mL	N/A		
	*Listeria monocytogenes*	MIC = 25 μg/mL	N/A		
	*Escherichia coli*	MIC = 500 μg/mL	N/A		
	*Yersinia enterocolitica*	MIC = 500 μg/mL	N/A		
	*Candida albicans* biofilm	MIC = 70 μg/mL	MIC = 0.06 μg/mL caspofungin	Garcinol prevented emergence of fungal germ tubes and showed cytostatic activity (via apoptosis), and thus inhibited development of hyphae and subsequent biofilm maturation	Jackson et al. (2015)
	*Helicobacter pylori*	IC_≥80_ = 100 μg/mL	IC_≥80_ = 0.5% vit. C IC_≥80_ = 100 μg/mL protykin, IC_≥80_ = 100 μg/mL garcinol + protykin 0.5% vit E – not active	Garcinol showed in vitro antimicrobial activity against *H. pylori*	Chatterjee et al. (2005)
In vitro anti-parasitical activity	*Toxoplasmosa gondii*	IC_50_ = 1.7 mg	N/A	Garcinol inhibited *Toxoplasma* tachyzoite replication and *P. falciparum* asexual replication (by inhibiting TgGCN5b, a nuclear GCN5 family KAT) with no toxicity to human host cells	Jeffers et al. (2016)
	*Plasmodium falciparum*	IC_50_ = 1.02—1.2 mg	N/A		

*p.o.* orally, *o.g.* oral gavage, *i.p.* intraperitoneally, *i.d.* intradermal application, *protykin* standardized extract of trans-resveratrol (20%) and emodin (10%)

#### Anti-inflammatory activity

Garcinol disposed an anti-inflammatory activity in various animal models of induced inflammation. Majority of studies agree on a mechanism that appears to be related to the interference with NF-κB, iNOS, ERK, COX-2, p38 mitogen-activated protein kinases (MAPK), lipoxygenase (5-LOX), TNF-α, interleukin (e.g. IL-2, IL-6, IL-23), nuclear factor of activated T-cells (NF-AT) (Liu et al. 2015; Schobert and Biersack 2019). Some authors also suggested that anti-inflammatory effect of garcinol is associated with HAT suppression (Ferriero et al. 2018).

#### Neurodegenerative disorders and drug withdrawal

Garcinol was found to be an inhibitor of monoamine oxidase B (MAO-B), and as such, it might be helpful in Parkinson’s disease treatment by retarding dopamine depletion (Mazumder et al. 2018). Additionally, it was discovered that garcinol attenuated the side-effects and increased bioavailability of L-DOPA, a dopamine precursor commonly used in the treatment of Parkinson’s disease symptoms (Mazumder et al. 2016; Ryu et al. 2018). Garcinol also decreased mortality and seizure scores in mice, presumably by suppressing brain-derived neurotrophic factor (BDNF) and by having effect on neurotransmitter systems, including those involving glutamate and GABA_A_ (Hao et al. 2016). Garcinol was also observed to decrease inflammation of microglia in rats via down regulation of NF-κB pathway and inhibiting COX-2, iNOS, and IL expression (Wang et al. 2017). A relatively unusual effect of garcinol has been discovered—in rats exposed to cocaine, garcinol inhibited restoration via reconsolidation-based modes following cocaine reactivation. The effect of garcinol on reactivated memories were long-lasting, suggesting a potential in control of drug abstinence and addiction (Fuchs and McLaughlin 2017).

#### Antiviral and antimicrobial activity

One of the early studies involved investigation on antiviral activity of garcinol against HIV, where again it was found to be potentially exerting its effect via inhibition of histone acetyltransferase (HAT) of the HIV infected cells (Mantelingu et al. 2007). Similarly as in the case of kolaviron, garcinol showed some degree of activity also against influenza virus (Hatakeyama et al. 2014). It appears that garcinol exerts its antiviral activity against influenza through regulation of the viral polymerase function (Schobert and Biersack 2019). Garcinol has demonstrated antibacterial, anti-yeast and antiprotozoal activity which was in some cases equal or better than conventional treatment. Again, mechanism of its antimicrobial effect might be related to the HAT inhibitory activity (noted above).

### Biological activities of garcinoic acid (GA)

Compared to KV and garcinol, there is only a limited number of studies on GA. Its biological activities are summarized in Table 7. In the early reports, GA showed in vitro antioxidant (Terashima et al. 2002; Okoko 2009) and anticancer effect (Mazzini et al. 2009; Birringer et al. 2010). GA was also found to improve heart function in myocardial infarction rats by increasing levels of pro-angiogenic factors, including hypoxia-inducible factor 1-alpha (HIF-1α), VEGF-A, and basic fibroblast growth factor (bFGF) (Hu et al. 2020). Very recently, garcinoic acid, together with related structures garcinal and tocotrienol, have showed phosphodiesterase-5 (PDE-5) inhibitory activity in molecular docking study (Ojo et al. 2021). Their activity was comparable to sildenafil (Viagra®), suggesting that these compounds may be useful in treatment of erectile disfunction. Additionally, GA was suggested to be of value as an agent with anti-inflammatory activity (Kluge et al. 2016). However, the exact mechanism of its anti-inflammatory action has not yet been entirely established, though it was indicated that GA may act as a COX-2 and iNOS inhibitor (Wallert et al. 2019). GA also reduced deposition of Aβ particles in brain in mouse a model of Alzheimer’s disease (Marinelli et al. 2020). Once again, human clinical studies of GA are not yet available.

**Table 7:** Biological activities of garcinoic acid

Disease/model	Animal/organ	Efficacious dose/mode of administration	Positive control	Major findings	References
*Cardiovascular disorders*					
Antiatherosclerotic effect	High fat diet fed ApoE ^−^/^−^ mice	1 mg/kg i.p	N/A	GA diminished lipopolysaccharide-induced upregulation of iNOS and COX-2 expression, decreased intra-plaque inflammation and changed NK and CD4 positive cell populations	Wallert et al. (2019)
*Inflammation and pain*					
Carrageenan-induced oedema model	Rats	50 mg/kg (N/A)	Indomethacin (10 mg/kg)	GA showed anti-inflammatory activity at comparable level to positive control	Tchimene et al. (2015a)
Anaesthesia	Guinea pigs	0.33, 0.66, 1.00 mg/kg i.d	Xylocaine (0.33, 0.66, 1.00 mg/kg)	GA induced local anaesthesia at comparable levels to xylocaine	Tchimene et al. (2015b)
Suppression of SARS-CoV-2 spike glycoprotein S1-induced hyper-inflammation	Human PBMC cells	0.5, 1.1, 2.1 µg/mL	N/A	GA reduced SARS-CoV-2 spike protein S1-induced secretion of TNFα, IL-6, IL-1β, and IL-8 in PBMCs	Olajide et al. (2021)
*Cancer*					
Brain	Glioma C6 cancer cells	4.3 µg/mL	N/A	GA showed in vitro antiproliferative effect on glioma C6 cancer cells	Mazzini et al. (2009)
*Central nervous system*					
Alzheimer’s disease	mice	5, 10, 25 mg/kg p.o	N/A	GA reduced Aβ aggregation and accumulation in mouse cortical astrocytes	Marinelli et al. (2020)
In vitro antibacterial activity	*Porphyromonas gingivalis*	MIC = 13.4 µg/mL	N/A	GA exhibited antimicrobial activity against both of these microorganisms	Hioki et al. (2020)
	*Streptococcus sobrinus*	MIC = 13.4 µg/mL	N/A		

*p.o.* orally, *i.p.* intraperitoneally, *i.d.* intradermal application

### Biological activity of xanthones

As it is also noted above *G. kola* also contains quite large number of xanthone derivatives, which are also widely distributed throughout the higher plants. Other xanthone-producing species from the Clusiaceae family include *Hypericum*, *Calophyllum*, *Kielmeyera* and *Tovomita* (Terashima et al. 1999). These compounds are attracting a considerable research interest as they (e.g. various hydroxy- and methoxy-analogues) were reported to possess a wide range of biological activities, including anticancer, antimalarial, antimicrobial, anti-HIV, anticonvulsant, anticholinesterase, antioxidant, anti-inflammatory effect (Miladiyah et al. 2018; Ramakrishnan et al. 2021). It was also found out that xanthones may interfere with several enzymes, including α-glucosidase, acyl-CoA:cholesterol acyltransferase, aromatase intestinal P-glycoprotein, miRNA, protein kinase C, topoisomerase, and xanthine oxidase. Especially the hydroxyxanthones are thought to provide promising anticancer activity and their semi-synthetic variants are widely researched as anticancer drugs (Miladiyah et al. 2018). It was suggested that their anticancer effect may be connected to various mechanisms, including inhibition of cyclooxygenase-2 (COX-2), NF-κB pathway, cyclin-dependent kinases (Cdk), and by suppressing expression of vascular endothelial growth factor (VEGF) and metalloproteinases (Klein-Júnior et al. 2020). Since biological activities and pharmacology of xanthones are quite extensively given elsewhere (see references provided above) and they are not specific constituents of bitter kola and other *Garcinia* species, they are covered in this review only superficially. It must be noted though, that to the best of our knowledge, none of the xanthones have advanced into clinical use and are not medicinally used in treatment of any human disease. In addition to that, it appears that pharmacological properties in humans, including solubility, lipophilicity, dissociation constant, chemical and metabolic stability, permeability, transporters modulation, and plasma protein bindings are largely unknown for xanthones (Gomes et al. 2016). Even toxic effects are to a large extent unknown. Xanthones should be treated with caution as some of the very closely related compounds (e.g. aflatoxins) are known to produce pronounced toxicity to humans (Dewick 2009).

### Toxicology

No serious adverse effects have been indicated in any of the available studies regarding the pharmacological activities of bitter kola. Few toxicological studies have been carried out which estimated the LD_50_ of the bitter kola seeds to be as low as 5000 mg/kg b.w. (Okoye et al. 2014). Currently, the only perceived health risk of bitter kola is that consuming too much of the seeds can lead to fertility issues (Dogara et al. 2022). This may be related to the ability of the present compounds to alter sexual hormone levels through interaction with various enzymes (e.g. 5α-reductase). However, this activity have not been proved in a satisfactory manner (Kalu et al. 2016; Winner et al. 2016). Interestingly, it is in contradiction to the traditional aphrodisiac ethnomedicinal indication of *G. kola*. More toxicological studies and clinical trials of bitter kola and its compounds are required to elucidate this issue and to avoid any complications in connection with their possible clinical use.

## Discussion

Majority of the available studies and review articles perceive KV as the active principle of *G. kola*. It has been investigated in a wide range of animal models and in vitro biological activities scenarios (see section biological activities of KV), where it was concluded that KV displays promising results in nearly every area studied and that it behaves almost like a panacea. A significant number of research articles are based on observing protective properties of KV against some toxin-induced disease model (e.g. kidney, liver, brain, heart, reproductive organs). Apart from very few exceptions, available studies have been using doses of KV (> 100 mg/kg, 200 mg/kg and in some cases even > 400 mg/kg), which may generally be viewed as excessive and from the clinical perspective unrealistic (calculated on a human body weight of 70 kg, the dose would correspond to administrations of approx. 7–14 g or even higher dose of pure substance). Even in the in vitro tests, it appears that in many cases way to high doses have been used (> 10 µg/mL). Problematics of using too high doses in animal and in vitro studies is extensively reviewed in Gertsch (2009). When using such large doses in animal models, another question arises, and that is whether apart from beneficial effects one would also expect to observe adverse effects. No studies so far focused on possible side effects induced by overdosing of KV. However, from the available data from structurally related polyphenolics (e.g. resveratrol), it appears that high doses (e.g. approx. 2.5 g of pure substance consecutively for few days) are associated with nausea, vomiting, diarrhoea and liver dysfunction (Salehi et al. 2018). Additionally, in many studies, only one dose of KV has been tested, which does not allow an insight into how the substance behaves in a dose-dependent manner and it does not provide statistical significance of a particular dose. Another problem with efficiency of KV lies in that very few studies have used appropriate control (many did not use positive control at all), and when they did so, it was used in an incomparable manner to the KV dose (often approx. 100-fold lower than that of KV). On the other hand, KV was in majority of studies administrated orally which corresponds with the traditional ethnomedicinal application.

Amentoflavone is another biflavonoid present in *G. kola*, whose research is even more extensive than that of KV (having more than 1000 references in scientific databases), yet it is very seldomly being associated with pharmacological properties of the species. Similarly, as in the case of KV, amentoflavone has been tested in many areas of pharmacological activity. In comparison to KV, however, majority of the available data on biological activities stems from studies using cells in vitro models, while studies based on observations in animals in vivo remains very limited. Contrastingly, it appears that the vast majority of animal studies involved reasonable doses of amentoflavone (10–100 mg/kg) (Yu et al. 2017; Xiong et al. 2021). Additionally, most of the in vivo studies used positive control in comparable doses to amentoflavone (Chen et al. 2018; Cao et al. 2021). However, both the tested substance, as well as the positive control were in many cases administrated via different route than orally (e.g. intraperitoneally, subcutaneously) (Kim et al. 1998; Shin et al. 2006; Sakthivel and Guruvayoorappan 2013; Zhao et al. 2017, 2019; Chen et al. 2018; Liu et al. 2020; Rizk et al. 2021), indicating, that amentoflavone has a poor pharmacokinetics. On top of that, these routes do not correspond with the traditional application of *G. kola*. In terms of toxicity, amentoflavone is relatively well studied, and demonstrated inhibition towards several important enzymes of the human cytochrome P-450, including CYP 1A2, 2A6, 2B6, 2D6, 2C, 2E1 and 3A. The strongest inhibition was observed in the case of CYP2C8 and 2C9, where the IC_50_’s were at 0.05 µg/mL (0.018 µM) and 0.08 µg/mL (0.15 µM), respectively. Other enzymes were inhibited in the range of 0.7–6.4 µg/mL (1.3–11.9 µM) (Park et al. 2020). Additionally, amentoflavone was also found to inhibit numerous UDP-glucuronosyl transferases 0.06–9.08 µg/mL (range 0.12–16.86 µM) (Lv et al. 2018). Interaction with CYP-450 is associated with altered activity of some prescription drugs (e.g. St. John’s Worth is known to interfere with such drugs as oral contraceptives, warfarin, digoxin, theophylline, indinavir, and cyclosporin) (Dewick 2009). Therefore, amentoflavone could be considered as an agent that potentially hinders activity of commonly prescribed drugs. Similar phenomenon have been observed for flavonoids associated with grapefruit (e.g. naringenin) (Fuhr et al. 1993). As far as we known, interference with CYP450 and UDP-glucuronosyl transferases was only observed for amentoflavone and remains unknown for other biflavonoids found in *G. kola*.

The research on volkensiflavone/morelloflavone shares many similarities with that of amentoflavone, except significantly lower number of studies on these compounds exist. However, it is again mainly restricted to in vitro studies. The available animal studies usually use only one dose (which in considerable share of studies is not based on application of pure compound but constitutes of mixture of structurally related compounds; for details see Table 5) and the administration route only involves intraperitoneal application. On top of that, as far as we know, positive control was used only in few studies. Even studies performed under in vitro seldomly uses it. In addition, from the clinical perspective, some of the studies present unrealistically high inhibitory concentrations (e.g. anti-cancer effect at levels 49.5 µg for morelloflavone and 100 µg/mL for amentoflavone) (Baggett et al. 2005), given that the in vitro efficiency of commonly used drugs (e.g. Taxol®) is in the range of ng/mL (nM) levels (Altmann and Gertsch 2007). Pharmacological efficiency of volkensiflavone/morelloflavone is thus questionable.

To the best of our knowledge, there are only two studies dealing with biological activity of garcinianin (Ajayi et al. 2014; Ito et al. 1999). It was tested for antibacterial effect against various pathogenic bacteria. Since garcinianin is found in roots and these are traditionally used as chewsticks for oral hygiene, this biological activity perfectly follows the ethnomedicinal indication. Garcinianin was found to be active against *Streptococcus mutans*, however, at enormously high dose (MIC = 1.0 mg/mL), being some 1000 times higher than commonly used antibiotics (Rubin et al. 2011). Additionally, it also showed potential anti-tumour promoting activity by inhibiting activity against 12-*O*-tetradecanoylphorbol-13-acetate (TPA)-induced Epstein-Barr virus early antigen activation in Raji cells at quite low concentrations (see Table 8).

**Table 8:** Biological activities of garcinianin

Disease/model	Animal/organ	Efficacious dose/mode of administration	Positive control	Major findings	References
Anti-tumour promoting activity	Inhibitory activity against 12-*O*-tetradecanoylphorbol-13-acetate (TPA)-induced Epstein-Barr virus early antigen activation in Raji cells	0.002–0.009 µg/mL	N/A	Garcinianin showed inhibitory activity against TPA-induced Epstein−Barr virus early antigen activation	Ito et al. (1999)
Antibacterial activity	*Streptococcus mutans*	1 mg/mL	Gentamicin (N/A)	Garcinianin showed in vitro antimicrobial activity against the corresponding bacteria	Ajayi et al. (2014)

Biological activity of *G. kola* biflavonoids is often being linked to the antioxidant-related mechanism (e.g. free radical scavenging activity, increased endogenous antioxidant defences, such as catalase, superoxide dismutase, and glutathione *S*-transferase). However, dietary antioxidants (including common flavonoids) have mostly failed to provide preventative and therapeutic activity in clinical studies of human disease, as reviewed in Halliwell (2012). The reasons behind flavonoids’ inactivity may lie in their high hydrophobicity, and resulting low bioavailability. On top of that, these molecules are present in nearly every higher plant, including food plants. Humans are thus heavily exposed to these compounds, which may have resulted in development of efficient metabolization and elimination of these compounds from our bodies (Tauchen et al. 2020). There are few examples of dietary antioxidants which are believed to provide therapeutic benefit in oxidative stress-related diseases. One example of such compound is ergothioneine, which occurs in food sources in a relatively small quantities, yet human body accumulates it efficiently in various tissues. Ergothioneine is transported to the sites of accumulation via very specific transporter OCTN1. During illness (such as neurodegenerative, eye, and cardiovascular disorders) ergothioneine blood levels are significantly decreased suggesting that a deficiency could be relevant to the disease onset or progression. None of these features have been thus far observed for flavonoids (Halliwell et al. 2018; Cheah and Halliwell 2021). Apart from antioxidant-related mode of action, *G. kola* biflavonoids have also been suggested to interfere with other systems, such as those involving inhibition of COX, phospholipase A2, aromatase, PDE, AChE, MAO-A, HMG-CoA reductase enzymes (see section biological activity of particular compound). For example, amentoflavone inhibits COX-1 and PDE at levels of 6.7 µg/mL (12.4 µM) (Bucar et al. 1998) and 0.15 µg/mL (0.27) (Saponara and Bosisio 1998), respectively. No inhibition was observed in the case of COX-2. However, in comparison to indomethacin 0.02 µg/mL (0.05 µM), amentoflavone shows some 250-fold COX-1 affinity (Kalgutkar et al. 2000) and to Viagra® approx. ten-fold PDE affinity (3.1 ng/mL; 6.6 nM) (Saenz De Tejada et al. 2001). Morelloflavone was shown to inhibit phospholipase A2 at IC_50_ = 0.9 µM (Gil et al. 1997), aromatase at 3.1 µM (Recalde-Gil et al. 2019), MAO-A at 5.1 µM (Recalde-Gil et al. 2017), and HMG-CoA at 80.9 µM (Tuansulong et al. 2011), which are again levels incomparable to commonly used drugs—darapladib (8.6 nM) (Hu et al. 2015), anastrazole (Arimidex®; 15 nM) (Miller 2006), harmaline (2.3 nM) (Kilpatrick et al. 2001), and mevastatin (23 nM) (Lin et al. 2015). Since all *G. kola* biflavonoids are structurally related, similar affinities may be expected for all of them. The exact mechanism of action of these compounds (if there is any clinically relevant) still remains unknown. Of particular importance is also to note that many flavonoids have been marked as pan-assay interfering compounds (abbreviated as PAINs), providing false positive results in many enzymic assays, by virtue of their chemistry (e.g. inhibiting enzymes not by specific mechanism, but via production of radicals, such as H_2_O_2_) (Bajorath 2021). Only very small number of flavonoid structures have successfully advanced to clinical use—examples of such compounds are intravenous silymarin in treatment of liver damage and injury (Ferenci 2016) and oral daflon® (mixture of micronized fraction of flavonoids, chiefly composed of 90% diosmin) (Lyseng-Williamson and Perry 2003). This indicates, that clinical efficiency of many flavonoids and their role in drug discovery remains questionable.

Numerous in vitro and in vivo studies about biological activity of garcinol were conducted over the last few years. In comparison to some other compounds of *G. kola*, garcinol is usually used in reasonable doses. On the other hand, most of the studies lack the comparison with positive control. Moreover, in vivo studies often do not respect traditional way of administration. Its synergistic effect with conventional anticancer agents when administrated orally belongs to the most convincing results. For example 0.05% garcinol in diet improved the response of transgenic pancreatic cancer mice to conventional treatment with gemcitabine from 10–15 to 25% (Saadat et al. 2018), Its combination with low dose of Taxol® was also able to better control the development of advanced or metastatic breast cancer (Tu et al. 2017). Additionally, it ameliorated the obesity-induced colon cancer, in this case, however, the i.p. application was better than the oral one. Garcinol also demonstrated in vitro antimicrobial activity against various G+ bacteria on the same levels as conventionally used antibiotics. The effect against G- bacteria is significantly weaker and in case of *E. coli* it is even contradictory (MIC 25 μg/mL vs. 500 μg/mL) (Table 6). Considering its significant activity against MRSA *S. aureus* strains, together with its potential to reduce the skin inflammation, the use of garcinol in topical treatments could become one of the research lead for this compound. A lot of evidence has been collected about garcinol positive effect against development and symptoms of Parkinson’s disease (Deb et al. 2019). It reduced seizure scores, mortality rates and improved memory of PD mice in the same doses as valproate (Hao et al. 2016). Furthermore, its effect on dyskinesia of mice when administered orally was comparable to other natural HAT inhibitors as anacardic acid and curcumin which were administered i.p. and in up to 50 times higher doses (Ryu et al. 2018). Garcinol was shown to inhibit HAT at IC_50_ of approx. 7 μM. (Balasubramanyam et al. 2004). The most potent reported HAT inhibitors identified so far are the bi-substrate inhibitors (e.g. H3-CoA-20; approx. IC_50_ = 300 nM) (Lau et al. 2000). Another detail which may point to garcinol being possibly of value as a pharmaceutical agent is its striking structural resemblance to some already established drugs, such as hyperforin from St. John’s Worth with antidepressant activity (Dewick 2009). However, despite some interesting research results about garcinol activity against PD and cancer, its pharmacokinetic properties have not been investigated in animal models yet. Therefore, its way to human clinical trials remains (at least for now) closed.

The research on garcinoic acid is very limited and chiefly constitutes of in vitro studies. Some of the presented effective doses, e.g. in the case of anticancer effect (Mazzini et al. 2009) are in the range (≈ 4.3 µg/mL; 10 µM) incomparable to conventionally used drugs (such as Taxol®, being efficient in nanomolar levels in in vitro tests) (Altmann and Gertsch 2007). The available animal studies use reasonable doses, though some are missing positive control (see Table 7). Even in majority of in vitro studies positive controls are not involved. As far as we know, only one study used oral administration (Marinelli et al. 2020). The remaining studies applied the compound via intraperitoneal or intradermal route, which is again not in correspondence with the traditional way of application. Number of studies dealing with garcinal is even more limited than in the case of garcionic acid and they are exclusively focused on determination of its antioxidant effect in vitro (Terashima et al. 1997, 2002). Since both garcinoic acid and garcinal are closely related to vitamin E, it has been suggested that they may provide therapeutic benefit through same antioxidant-related mechanism. However, it has been implied that mode of action of vitamin E may be derived from production of cell signaling and specific regulation of various genes rather than antioxidant activity (Azzi and Zingg 2005). This may also be true in the case of garcinoic acid and garcinal. Additionally, it has been previously shown that the above-mentioned biological effect is quite unique for α-tocopherol and the activity of related structures (e.g. β-, γ-, and δ-tocopherol) is significantly weaker (being 50%, 10%, and 3%, respectively) (Dewick 2009). This also applies for tocotrienols. It is therefore uncertain whether *G. kola* derived vitamin E derivatives have the capability to produce comparable pharmacological effect as α-tocopherol or their efficiency is significantly diminished as in the case of remaining α-tocopherol derivatives. Additionally, δ-tocotrienol, garcinoic acid and garcinal have displayed similar affinity towards PDE-5 as Viagra®, however, these results were only thus far observed in an in silico model (Ojo et al. 2021). Vitamin E have largely failed in clinical trials to provide therapeutic benefits in various human diseases (such as cardiovascular disorders, hypertension, diabetes, and cancer) (Robinson et al. 2006; Steinhubl 2008). Though generally recognized as safe, it has been found that high doses of vitamin E are associated with manifestation of various side effects, including hemorrhagic stroke (Sesso et al. 2008) and increased risk of prostatic cancer (Klein et al. 2011). In addition, vitamin E was suggested to interact with cytochrome P-450-dependent drug-metabolizing system (Brigelius-Flohé 2007), thus giving a one possible explanation to its ability to increase the blood thinning activity of warfarin (Fan et al. 2017). Quite recently, garcinoic acid was found to interfere with pregnane X receptor, which leads to regulation of cytochrome P-450 system (Bartolini et al. 2020). It thus appears, that apart from other adverse effects mentioned above, vitamin E and related structures (including garcinoic acid) may jeopardize therapeutic efficiency of some commonly used drugs.

Kolanone was mainly tested in in vitro antibacterial activity assays. As far as we known, only one study used isolated compound (Hussain et al. 1982); the remaining studies used extracts which under subsequent chemical analysis were found to contain kolanone. However, other compounds could also contribute to the observed antimicrobial effect. Again, there is a rationale behind testing kolanone for antibacterial effect, since it is also present in roots which are used as chewing sticks for oral hygiene. For the determination of antibacterial activity, Hussain et al. (1982) have used disc-diffusion methods and observed zones of inhibition at 14–15 mm for *Pseudomonas aeruginosa*, *Staphylococcus aureus*, *Bacillus subtilis*, *Streptococcus pneumoniae*, and *Candida albicans*. However, kolanone was applied in a solution of a very high concentration (1% w/v) without comparing its activity to proper positive control. Furthermore, the diffusion method is not appropriate for testing non-polar samples or those that do not easily diffuse into agar (Cos et al. 2006). Since kolanone appears to be derived from polyketide metabolism and is seemingly of non-polar nature, it may not be suitable for this kind of method. Recently, kolanone was tested in the rat model of ethanol-HCl-induced gastric ulcers (Uwagie-Ero et al. 2020). It was administered in various doses (25, 50 and 75 mg/kg) and its efficiency was comparable to omeprazole (20 mg/kg). However, the mode of administration was not indicated. To the best of our knowledge, there is no study on the biological activity of gakolanone, a structurally related compound to kolanone. Since both kolanone and gakolanone are quite unusual constituents, and only limited share of studies were focused on them, questions may be raised about the veracity of deriving their structure. However, the structure was elucidated through UV, IR, MS, NMR techniques (Hussain et al. 1982; Akoro et al. 2020). Kolanone was also independently semi-synthetized in laboratory conditions (Raikar et al. 2008) (see Table 9).

**Table 9:** Biological activities of kolanone

Disease/model	Animal/organ	Efficacious dose/mode of administration	Positive control	Major findings	References
Antiulcer activity	Mice	25, 50, 75 mg/kg (N/A)	omeprazole (20 mg/kg)	Kolanone showed gastro-protective effect against ethanol-induced stomach ulcers and reduced ethanol-induced lipid peroxidation	Uwagie-Ero et al. (2020)
In vitro antibacterial activity (zone inhibition test)	*Bacillus subtilis, Pseudomonas aeruginosa*, *Staphylococcus aureus*, *Streptococcus pneumoniae*, *Candida albicans*	10 µg/mL (zone of inhibition = 14–15 mm)	N/A	Kolanone exhibited antimicrobial activity against the corresponding bacteria	Hussain et al. (1982)

For the remaining substances, garcifuran A and B, and garcipyran, the biological activity remains unknown. Only phytochemical records exist of these substances, and all of them have been thus far exclusively found only in *G. kola* (Niwa et al. 1994a, b). Again, as they represent a quite unique structure, and only very limited number of studies addressed them, concerns may be raised if their structures have been elucidated accurately. As in the case of kolanone and gakolanone, structures of both garcifurans and garcipyran have been elucidated via MS, IR, UV, and NMR. On top of that, there is one study reporting total synthesis of garcipyran B (Kelly et al. 1997). Pharmacological efficiency of these compounds may be questioned as well. However, some of the simple, as well as more complex, benzofuran derivatives are potentially of value as medicinal agents or are already in clinical use (such as griseofulvin, methoxalen, amiodarone, benziodarone, dimemebfe, efaroxan, elopiprazole).

*Garcinia kola* also contains phytosterols cycloartenol and 24-methylenecycloartenol, though they are quite abundant in the nature and are also found in other species including such genera as *Artocarpus*, *Euphorbia*, *Costus*, *Polygonum*, and *Schinziophyton*. In a recent study by Sadasivan Nair et al. (2020), both cycloartenol and 24-methylenecycloartenol showed glucose lowering activity in an oral glucose tolerance test in high fat diet-streptozotocin induced type II diabetic rats. Their antidiabetic activity might stem from ability to inhibit α-glucosidase (Nokhala et al. 2020). 24-Methylenecycloartenol was also found to attenuate acetic acid-induced pain in mice models of nociception (Ferreira et al. 2000). It also produced anti-inflammatory, antibacterial, and antiplasmodial effect (Akihisa et al. 1996; Bickii et al. 2006; Ajayi et al. 2014). Though showing some activities, both cycloartenol and 24-methylenecycloartanol are regarded as the starting structures and intermediates for the biosynthesis of other biologically active molecules (e.g. phytostanols, phytosterols) and are generally not considered as pharmacologically important compounds.

On top of so far discussed compounds, *G. kola* also contains exogenous constituents collectively referred to as cytochalasins, specifically 8-metoxycytochalasin J, cytochalasin H, cytochalasin J and alternariol, which appears to be product of the endophytic fungi (*Phomosis* sp.) associated with the seed but are not synthesized by the plant itself. These compounds have been tested for antibacterial effect against various microorganisms, including *Vibrio cholerae*, *Shigella flexneri*, and *Staphylococcus aureus* and cytotoxic activity against HeLa cells, thought their efficiency was quite low (MIC ranged from 128 to 512 µg/mL and IC_50_ against HeLa cells was in the range 0.25–35.69 µg/mL) (Jouda et al. 2016). Again, these activities are incomparably high to commonly used antibiotics and anticancer drugs (for references see above). Cytochalasin are quite recently discovered compounds. Their pharmacological value remains to be established.

As it is noted on several occasions in this review, KV is largely perceived as the active principle of *G. kola*. However, from what we know so far, other compounds present in *G. kola*, such as garcinol, garcifuran A and B, kolanone and gakolanone, may largely contribute to the bioactivities of *G. kola* and perhaps administer a greater promise for the drug discovery. However, none of the compounds found in *G. kola* have been subjected to the human clinical trials as of yet. Without them, any statement about what substance(s) is/are responsible for the biological activity of *G. kola* is a mere speculation. Although some compounds may display promising in vitro and in vivo activity, these results are unfortunately to a large extent not transferable to the clinical environment (as many compounds that were found to be effective in animal models later failed to provide sufficient action in humans) (Bracken 2009; Gertsch 2009), and this may also be the case of *G. kola* derived compounds.

There is a strong indication that *G. kola* possess some therapeutic benefits, as documented by its widespread use in folk medicine. Despite the numerous studies that have been conducted on bitter kola compounds, we still have little definite evidence of which substances are responsible for these therapeutic effects, nor do we know their exact mechanism of action. It is also quite possible that previously unknown substances are responsible for the biological activities of the species. There might be an analogy with turmeric (*Curcuma longa*), a traditional medicinal plant of Ayurveda, where curcumin has been identified as the active principle, yet available clinical studies have shown contradictory results. It appears that other, thus far unidentified compounds are responsible for the therapeutic benefit of the plant (Baker 2017).

## Concluding remarks

*Garcinia kola* is an important medicinal plant with a long history of being used in the treatment of a wide range of human diseases. It contains several very specific compounds, which may be responsible for the observed biological activity and pharmacological properties of this plant. However, biological activity of these compounds, including perhaps the most studied substance kolaviron, has been only studied in animals. Confirmation that these substances are responsible for the therapeutic effects of the *G. kola* must be based on sufficiently powerful, double-blind, placebo-controlled clinical studies in humans (together with elucidation of their modes of action, therapeutic dose, adverse-effect profile, and other pharmacological data), which are unfortunately to date unavailable. We are afraid that at this moment therapeutic efficacy of any compound present in *G. kola* is far from conclusive. In connection to that, due to the relatively wide portfolio of diseases that are traditionally treated with *G. kola* and an even greater number of biological activities demonstrated by the present compounds, it is still impossible to reliably identify a substance that could be associated with the traditional ethnomedical use of *G. kola*. Many review articles have identified kolaviron as the active principle of *G. kola*. Perhaps garcinol, due to the relatively promising pharmacological activity (e.g. anticancer, antimicrobial, neuroprotective activities) deserves a deeper scientific interest. However, it is also likely that the substances potentially responsible for the pharmacological properties of the bitter kola have not yet been discovered. It is also possible that the constituents in *G. kola* work in synergy and, when isolated, will not provide such results as in the form of complex mixture in the natural material (as for example seen in the case of rauwolfia alkaloids). Hopefully some human clinical trials will be performed with the extracts/compounds from *G. kola* in the future and a promising candidate will emerge with the potential of becoming an important lead for the drug development.

## References

[CR1] Abarikwu SO (2014). Anti-inflammatory effects of kolaviron modulate the expressions of inflammatory marker genes, inhibit transcription factors ERK1/2, p-JNK, NF-κB, and activate Akt expressions in the 93RS2 Sertoli cell lines. Mol Cell Biochem.

[CR2] Abarikwu SO, Farombi EO, Kashyap MP, Pant AB (2011). Kolaviron protects apoptotic cell death in PC12 cells exposed to Atrazine. Free Radic Res.

[CR3] Abarikwu SO, Farombi EO, Pant AB (2011). Biflavanone-kolaviron protects human dopaminergic SH-SY5Y cells against atrazine induced toxic insult. Toxicol In Vitro.

[CR4] Abarikwu SO, Njoku R-CC, John IG (2021). Antioxidant and anti-inflammatory protective effects of rutin and kolaviron against busulfan-induced testicular injuries in rats. Syst Biol Reprod Med.

[CR5] Abdallah HM, Almowallad FM, Esmat A (2015). Anti-inflammatory activity of flavonoids from *Chrozophora tinctoria*. Phytochem Lett.

[CR6] Acuña UM, Dastmalchi K, Basile MJ, Kennelly EJ (2012). Quantitative high-performance liquid chromatography photo-diode array (HPLC-PDA) analysis of benzophenones and biflavonoids in eight Garcinia species. J Food Compos Anal.

[CR7] Adaramoye OA (2009). Comparative effects of vitamin E and kolaviron (a biflavonoid from *Garcinia kola*) on carbon tetrachloride-induced renal oxidative damage in mice. Pak J Biol Sci.

[CR8] Adaramoye OA (2012). Antidiabetic effect of kolaviron, a biflavonoid complex isolated from *Garcinia kola* seeds, in Wistar rats. Afr Health Sci.

[CR9] Adaramoye OA, Adeyemi EO (2006). Hypoglycaemic and hypolipidaemic effects of fractions from kolaviron, a biflavonoid complex from *Garcinia Kola* in streptozotocin-induced diabetes mellitus rats. J Pharm Pharmacol.

[CR10] Adaramoye OA, Arisekola M (2012). Kolaviron, a biflavonoid complex from *Garcinia kola* seeds, ameliorates ethanol-induced reproductive toxicity in male wistar rats. Niger J Physiol Sci.

[CR11] Adaramoye OA, Nwaneri VO, Anyanwo KC (2005). Possible anti-atherogenic effect of kolaviron (a *Garcinia kola* seed extract) in hypercholesterolaemic rats. Clin Exp Pharmacol Physiol.

[CR12] Adaramoye OA, Farombi EO, Nssien M (2008). Hepatoprotective activity of purified fractions from *Garcinia kola* seeds in mice intoxicated with carbon tetrachloride. J Med Food.

[CR13] Adaramoye OA, Akanni OO, Farombi EO (2013). Nevirapine induces testicular toxicity in wistar rats: reversal effect of kolaviron (biflavonoid from *Garcinia kola* seeds). J Basic Clin Physiol Pharmacol.

[CR14] Adaramoye OA, Kehinde AO, Adefisan A (2016). Ameliorative effects of kolaviron, a biflavonoid fraction from *Garcinia kola* seed, on hepato-renal toxicity of anti-tuberculosis drugs in wistar rats. Tokai J Exp Clin Med.

[CR15] Adedara IA, Farombi EO (2012). Chemoprotection of ethylene glycol monoethyl ether-induced reproductive toxicity in male rats by kolaviron, isolated biflavonoid from *Garcinia kola* seed. Hum Exp Toxicol.

[CR16] Adedara IA, Farombi EO (2013). Chemoprotective effects of kolaviron on ethylene glycol monoethyl ether-induced pituitary-thyroid axis toxicity in male rats. Andrologia.

[CR17] Adedara IA, Farombi EO (2014). Kolaviron protects against ethylene glycol monoethyl ether-induced toxicity in boar spermatozoa. Andrologia.

[CR19] Adedara IA, Owoeye O, Aiyegbusi MA (2015). Kolaviron protects against benzo[a]pyrene-induced functional alterations along the brain-pituitary-gonadal axis in male rats. Environ Toxicol Pharmacol.

[CR20] Adedara IA, Awogbindin IO, Maduako IC (2021). Kolaviron suppresses dysfunctional reproductive axis associated with multi-walled carbon nanotubes exposure in male rats. Environ Sci Pollut Res.

[CR21] Adesuyi AO, Elumm IK, Adaramola FB, Nwokocha AGM (2012). Nutritional and phytochemical screening of *Garcinia kola*. Adv J Food Sci Technol.

[CR22] Adewole KE, Gyebi GA, Ibrahim IM (2021). Amyloid β fibrils disruption by kolaviron: molecular docking and extended molecular dynamics simulation studies. Comput Biol Chem.

[CR23] Adewole KE, Ishola AA, Omolaso BO (2021). Identification of potential histone deacetylase inhibitory biflavonoids from *Garcinia kola* (Guttiferae) using in silico protein-ligand interaction. Phys Sci Rev.

[CR24] Adoga JO, Channa ML, Nadar A (2021). Kolaviron attenuates cardiovascular injury in fructose-streptozotocin induced type-2 diabetic male rats by reducing oxidative stress, inflammation, and improving cardiovascular risk markers. Biomed Pharmacother.

[CR25] Agboola OS, Oyagbemi AA, Omobowale TO (2016). Modulatory role of Kolaviron (KV), a biflavonoid from Garcinia kola, in sodium arsenite-induced hepatotoxicity and haematotoxicity in rats. Toxicol Int.

[CR26] Aggarwal V, Tuli HS, Kaur J (2020). Garcinol exhibits anti-neoplastic effects by targeting diverse oncogenic factors in tumor cells. Biomedicines.

[CR27] Ainslie JR (1937). List of plants used in native medicine in Nigeria.

[CR28] Ajani EO, Shallie PD, Adegbesan BO (2008). Protective effect of *Garcinia Kola* (Kolaviron) extract on predisposition of rats to cardiovascular diseases following separate administration of amodiaquine and artesunate. Afr J Tradit Complement Altern Med.

[CR29] Ajayi TO, Moody JO, Fukushi Y (2014). Antimicrobial activity of *Garcinia kola* (Heckel) seed extracts and isolated constituents against caries-causing microorganisms. Afr J Biomed Res.

[CR30] Akihisa T, Yasukawa K, Oinuma H (1996). Triterpene alcohols from the flowers of compositae and their anti-inflammatory effects. Phytochemistry.

[CR31] Akinmoladun AC, Saliu IO, Olowookere BD (2018). Improvement of 2-vessel occlusion cerebral ischaemia/reperfusion-induced corticostriatal electrolyte and redox imbalance, lactic acidosis and modified acetylcholinesterase activity by kolaviron correlates with reduction in neurobehavioural deficits. Ann Neurosci.

[CR32] Akinrinde AS, Olowu E, Oyagbemi AA, Omobowale OT (2015). Gastrointestinal protective efficacy of Kolaviron (a bi-flavonoid from *Garcinia kola*) following a single administration of sodium arsenite in rats: biochemical and histopathological studies. Pharmacogn Res.

[CR33] Akinrinde AS, Omobowale O, Oyagbemi A (2016). Protective effects of kolaviron and gallic acid against cobalt-chloride-induced cardiorenal dysfunction via suppression of oxidative stress and activation of the ERK signaling pathway. Can J Physiol Pharmacol.

[CR34] Akoro SM, Aiyelaagbe OO, Onocha PA, Gloer JB (2020). Gakolanone: a new benzophenone derivative from *Garcinia kola* Heckel stem-bark. Nat Prod Res.

[CR35] Alabi QK, Akomolafe RO (2020). Kolaviron diminishes diclofenac-induced liver and kidney toxicity in wistar rats via suppressing inflammatory events, upregulating antioxidant defenses, and improving hematological indices. Dose-Response.

[CR36] Alabi QK, Akomolafe RO, Olukiran OS (2017). The *Garcinia kola* biflavonoid kolaviron attenuates experimental hepatotoxicity induced by diclofenac. Pathophysiology.

[CR37] Alabi QK, Akomolafe RO, Olukiran OS (2018). Kolaviron attenuates diclofenac-induced nephrotoxicity in male wistar rats. Appl Physiol Nutr Metab.

[CR38] Altmann K-H, Gertsch J (2007). Anticancer drugs from nature: natural products as a unique source of new microtubule-stabilizing agents. Nat Prod Rep.

[CR39] Awogbindin IO, Olaleye DO, Farombi EO (2015). Kolaviron improves morbidity and suppresses mortality by mitigating oxido-inflammation in BALB/c mice infected with influenza virus. Viral Immunol.

[CR40] Awogbindin IO, Olaleye DO, Farombi EO (2017). Mechanistic perspective of the oxido-immunopathologic resolution property of kolaviron in mice influenza pneumonitis. APMIS.

[CR41] Awogbindin IO, Maduako IC, Adedara IA (2021). Kolaviron ameliorates hepatic and renal dysfunction associated with multiwalled carbon nanotubes in rats. Environ Toxicol.

[CR42] Ayepola OR, Brooks NL, Oguntibeju OO (2014). Kolaviron improved resistance to oxidative stress and inflammation in the blood (erythrocyte, serum, and plasma) of streptozotocin-induced diabetic rats. Sci World J.

[CR43] Ayepola OR, Cerf ME, Brooks NL, Oguntibeju OO (2014). Kolaviron, a biflavonoid complex of *Garcinia kola* seeds modulates apoptosis by suppressing oxidative stress and inflammation in diabetes-induced nephrotoxic rats. Phytomedicine.

[CR44] Azebaze AGB, Teinkela JEM, Nguemfo EL (2015). Antiplasmodial activity of some phenolic compounds from cameroonians allanblackia. Afr Health Sci.

[CR45] Azzi A, Zingg J-M (2005). Vitamin E: textbooks require updating. Biochem Mol Biol Educ.

[CR46] Baggett S, Protiva P, Mazzola EP (2005). Bioactive benzophenones from Garcinia xanthochymus fruits. J Nat Prod.

[CR47] Bajorath J (2021). Evolution of assay interference concepts in drug discovery. Expert Opin Drug Discov.

[CR48] Baker M (2017). Deceptive curcumin offers cautionary tale for chemists. Nature.

[CR49] Balasubramanyam K, Altaf M, Varier RA (2004). Polyisoprenylated benzophenone, garcinol, a natural histone acetyltransferase inhibitor, represses chromatin transcription and alters global gene expression. J Biol Chem.

[CR50] Bartolini D, De Franco F, Torquato P (2020). Garcinoic acid is a natural and selective agonist of pregnane X receptor. J Med Chem.

[CR51] Baureithel KH, Büter KB, Engesser A (1997). Inhibition of benzodiazepine binding in vitro by amentoflavone, a constituent of various species of Hypericum. Pharm Acta Helv.

[CR52] Bezerra ÉA, Alves MMM, Lima SKR (2021). Biflavones from platonia insignis mart. Flowers promote in vitro antileishmanial and immunomodulatory effects against internalized amastigote forms of leishmania amazonensis. Pathogens.

[CR53] Bickii J, Tchouya GRF, Tchouankeu JC, Tsamo E (2006). The antiplasmodial agents of the stem bark of Entandrophragma angolense (Meliaceae). Afr J Tradit Complement Altern Med.

[CR54] Birringer M, Lington D, Vertuani S (2010). Proapoptotic effects of long-chain vitamin E metabolites in HepG2 cells are mediated by oxidative stress. Free Radic Biol Med.

[CR55] Bracken MB (2009). Why animal studies are often poor predictors of human reactions to exposure. J R Soc Med.

[CR56] Braide VB (1991). Antihepatotoxic biochemical effects of kolaviron, a biflavonoid of *Garcinia kola* seeds. Phytother Res.

[CR57] Brigelius-Flohé R (2007). Adverse effects of vitamin E by induction of drug metabolism. Genes Nutr.

[CR58] Brusotti G, Papetti A, Serra M (2016). *Allanblackia floribunda* Oliv.: an aphrodisiac plant with vasorelaxant properties. J Ethnopharmacol.

[CR59] Bucar F, Jackak SM, Noreen Y (1998). Amentoflavone from Biophytum sensitivum and its effect on COX-1/COX-2 catalysed prostaglandin biosynthesis. Planta Med.

[CR60] Cao B, Zeng M, Zhang Q (2021). Amentoflavone ameliorates memory deficits and abnormal autophagy in Aβ_25–35_-induced mice by mTOR signaling. Neurochem Res.

[CR61] Chatterjee A, Bagchi D, Yasmin T, Stohs SJ (2005). Antimicrobial effects of antioxidants with and without clarithromycin on Helicobacter pylori. Mol Cell Biochem.

[CR62] Cheah IK, Halliwell B (2021). Ergothioneine, recent developments. Redox Biol.

[CR63] Cheek M (2004) *Garcinia kola*. IUCN Red List Threat Species e.T34715A9884648

[CR64] Chen C, Li B, Cheng G (2018). Amentoflavone ameliorates Aβ_1–42_-induced memory deficits and oxidative stress in cellular and rat model. Neurochem Res.

[CR65] Chetia Phukan B, Dutta A, Deb S (2022). Garcinol blocks motor behavioural deficits by providing dopaminergic neuroprotection in MPTP mouse model of Parkinson’s disease: involvement of anti-inflammatory response. Exp Brain Res.

[CR66] Corona A, Seibt S, Schaller D (2021). Garcinol from Garcinia indica inhibits HIV-1 reverse transcriptase-associated ribonuclease H. Arch Pharm (Weinheim).

[CR67] Cos P, Vlietinck AJ, Berghe DV, Maes L (2006). Anti-infective potential of natural products: how to develop a stronger in vitro “proof-of-concept”. J Ethnopharmacol.

[CR68] Coulerie P, Nour M, Maciuk A (2013). Structure-activity relationship study of biflavonoids on the dengue virus polymerase DENV-NS5 RdRp. Planta Med.

[CR69] Deb S, Phukan BC, Mazumder MK (2019). Garcinol, a multifaceted sword for the treatment of Parkinson’s disease. Neurochem Int.

[CR70] Decha-Dier U, Hutadilok-Towatana N, Mahabusarakam W (2008). Anti-atherogenic effects of morelloflavone from *Garcinia dulcis* leaves in cholesterol fed rabbits. J Nat Remedies.

[CR71] Dewick PM (2009). Medicinal natural products: a biosynthetic approach.

[CR72] Dogara AM, Hamad SW, Hama HA (2022). Biological evaluation of *Garcinia kola* Heckel. Adv Pharmacol Pharm Sci.

[CR73] Dozie-Nwakile OC, Dozie NC, Kingsley UI (2021). Effects of kolaviron on pneumonia-like infection induced in albino wistar rats. Anti-Inflamm Anti-Allergy Agents Med Chem.

[CR74] E Silva AKF, Dos Reis AC, Pinheiro EEA (2021). Modulation of the drug resistance by platonia insignis mart. Extract, ethyl acetate fraction and morelloflavone/volkensiflavone (biflavonoids) in staphylococcus aureus strains overexpressing efflux pump genes. Curr Drug Metab.

[CR75] Eleazu CO, Eleazu KC, Awa E, Chukwuma SC (2012). Comparative study of the phytochemical composition of the leaves of five Nigerian medicinal plants. J Biotechnol Pharm Res.

[CR76] Eleyinmi AF, Bressler DC, Amoo IA (2006). Chemical composition of bitter cola (*Garcinia kola*) seed and hulls. Pol J Food Nutr Sci.

[CR77] El-Seedi HR, El-Ghorab DMH, El-Barbary MA (2009). Naturally occurring xanthones; latest investigations: isolation, structure elucidation and chemosystematic significance. Curr Med Chem.

[CR78] Emmanuel O, Uche ME, Dike ED (2022). A review on *garcinia kola* heckel: traditional uses, phytochemistry, pharmacological activities, and toxicology. Biomarkers.

[CR79] Erukainure OL, Salau VF, Chukwuma CI, Islam MS (2021). Kolaviron: a biflavonoid with numerous health benefits. Curr Pharm Des.

[CR80] Fan Y, Adam TJ, McEwan R (2017). Detecting signals of interactions between warfarin and dietary supplements in electronic health records. Stud Health Technol Inform.

[CR81] Farombi EO (2006). Genotoxicity of chloroquine in rat liver cells: protective role of free radical scavengers. Cell Biol Toxicol.

[CR82] Farombi EO, Tahnteng JG, Agboola AO (2000). Chemoprevention of 2-acetylaminofluorene-induced hepatotoxicity and lipid peroxidation in rats by kolaviron: a *Garcinia kola* seed extract. Food Chem Toxicol.

[CR83] Farombi EO, Adepoju BF, Ola-Davies OE, Emerole GO (2005). Chemoprevention of aflatoxin B1-induced genotoxicity and hepatic oxidative damage in rats by kolaviron, a natural biflavonoid of *Garcinia kola* seeds. Eur J Cancer Prev.

[CR84] Farombi EO, Abarikwu SO, Adedara IA, Oyeyemi MO (2007). Curcumin and kolaviron ameliorate di-n-butylphthalate-induced testicular damage in rats. Basic Clin Pharmacol Toxicol.

[CR85] Farombi EO, Shrotriya S, Surh Y-J (2009). Kolaviron inhibits dimethyl nitrosamine-induced liver injury by suppressing COX-2 and iNOS expression via NF-κB and AP-1. Life Sci.

[CR86] Farombi EO, Adedara IA, Akinrinde SA (2012). Protective effects of kolaviron and quercetin on cadmium-induced testicular damage and endocrine pathology in rats. Andrologia.

[CR87] Farombi EO, Abolaji AO, Farombi TH (2018). *Garcinia kola* seed biflavonoid fraction (Kolaviron), increases longevity and attenuates rotenone-induced toxicity in *Drosophila melanogaster*. Pestic Biochem Physiol.

[CR88] Farombi EO, Awogbindin IO, Farombi TH (2019). Neuroprotective role of kolaviron in striatal redo-inflammation associated with rotenone model of Parkinson’s disease. Neurotoxicology.

[CR89] Farombi EO, Awogbindin IO, Olorunkalu PD (2020). Kolaviron protects against nigrostriatal degeneration and gut oxidative damage in a stereotaxic rotenone model of Parkinson’s disease. Psychopharmacology.

[CR90] Farombi EO, Awogbindin IO, Owoeye O (2020). Kolaviron ameliorates behavioural deficit and injury to striatal dopaminergic terminals via modulation of oxidative burden, DJ-1 depletion and CD45R+ cells infiltration in MPTP-model of Parkinson’s disease. Metab Brain Dis.

[CR91] Ferenci P (2016). Silymarin in the treatment of liver diseases: what is the clinical evidence?. Clin Liver Dis.

[CR92] Ferreira J, Floriani AEO, Filho VC (2000). Antinociceptive properties of the methanolic extract and two triterpenes isolated from *Epidendrum mosenii* stems (Orchidaceae). Life Sci.

[CR93] Ferriero R, Nusco E, De Cegli R (2018). Pyruvate dehydrogenase complex and lactate dehydrogenase are targets for therapy of acute liver failure. J Hepatol.

[CR94] Fuchs RA, McLaughlin RJ (2017). Garcinol: a magic bullet of amnesia for maladaptive memories?. Neuropsychopharmacology.

[CR95] Fuhr U, Klittich K, Staib A (1993). Inhibitory effect of grapefruit juice and its bitter principal, naringenin, on CYP1A2 dependent metabolism of caffeine in man. Br J Clin Pharmacol.

[CR96] Gertsch J (2009). How scientific is the science in ethnopharmacology? Historical perspectives and epistemological problems. J Ethnopharmacol.

[CR97] Gil B, Sanz MJ, Terencio MC (1997). Morelloflavone, a novel biflavonoid inhibitor of human secretory phospholipase A2 with anti-inflammatory activity. Biochem Pharmacol.

[CR98] Gomes AS, Brandão P, Fernandes CSG (2016). Drug-like properties and ADME of xanthone derivatives: the antechamber of clinical trials. Curr Med Chem.

[CR99] Gwatidzo L, Botha BM, McCrindle RI, Combrinck S (2014). Extraction and identification of phytosterols in manketti (*Schinziophyton rautanenii*) nut oil. JAOCS J Am Oil Chem Soc.

[CR100] Halliwell B (2012). Free radicals and antioxidants: updating a personal view. Nutr Rev.

[CR101] Halliwell B, Cheah IK, Tang RMY (2018). Ergothioneine: a diet-derived antioxidant with therapeutic potential. FEBS Lett.

[CR102] Hao F, Jia L-H, Li X-W (2016). Garcinol upregulates GABAA and GAD65 expression, modulates BDNF-TrkB pathway to reduce seizures in pentylenetetrazole (PTZ)-induced epilepsy. Med Sci Monit.

[CR103] Hatakeyama D, Shoji M, Yamayoshi S (2014). A novel functional site in the PB2 subunit of influenza a virus essential for acetyl-CoA interaction, RNA polymerase activity, and viral replication. J Biol Chem.

[CR104] Hioki Y, Onwona-Agyeman S, Kakumu Y (2020). Garcinoic acids and a benzophenone derivative from the seeds of *Garcinia kola* and their antibacterial activities against oral bacterial pathogenic organisms. J Nat Prod.

[CR105] Hu C, Tompson D, Magee M (2015). Single and multiple dose pharmacokinetics, pharmacodynamics and safety of the novel lipoprotein-associated phospholipase A2 enzyme inhibitor darapladib in healthy Chinese subjects: an open label phase-1 clinical trial. PLoS ONE.

[CR106] Hu H, Zhao H, Wu Z, Rao M (2020). Effects of garcinoic acid on cardiac function and its mechanism in post-myocardial infarction rats. Med J Wuhan Univ.

[CR107] Huang W-C, Kuo K-T, Adebayo BO (2018). Garcinol inhibits cancer stem cell-like phenotype via suppression of the Wnt/β-catenin/STAT3 axis signalling pathway in human non-small cell lung carcinomas. J Nutr Biochem.

[CR108] Hung WL, Liu CM, Lai CS (2015). Inhibitory effect of garcinol against 12-O-tetradecanoylphorbol 13-acetate-induced skin inflammation and tumorigenesis in mice. J Funct Foods.

[CR109] Hussain RA, Owegby AG, Waterman PG (1982). Kolanone, a novel polyisoprenylated benzophenone with antimicrobial properties from the fruit of *Garcinia kola*. Planta Med.

[CR110] Hyun JJ, Woo SS, Yeo S-H (2006). Antifungal effect of amentoflavone derived from *Selaginella tamariscina*. Arch Pharm Res.

[CR111] Ibironke GF, Fasanmade AA (2015). Analgesic and central nervous system depressant activities of kolaviron (A *Garcinia kola* biflavonoid complex). Afr J Biomed Res.

[CR112] Ibironke GF, Fasanmade AA (2016). Neuroprotective effects of kolaviron against psycho-emotional stress induced oxidative brain injury in rats: the whisker removal model. Afr J Med Med Sci.

[CR113] Igado OO, Olopade JO, Adesida A (2012). Morphological and biochemical investigation into the possible neuroprotective effects of kolaviron (*Garcinia kola* bioflavonoid) on the brains of rats exposed to vanadium. Drug Chem Toxicol.

[CR114] Iinuma M, Tosa H, Tanaka T (1996). Antibacterial activity of some Garcinia benzophenone derivatives against methicillin-resistant Staphylococcus aureus. Biol Pharm Bull.

[CR115] Ijomone OM, Obi AU (2013). Kolaviron, isolated from *Garcinia kola*, inhibits acetylcholinesterase activities in the hippocampus and striatum of Wistar rats. Ann Neurosci.

[CR116] Ijomone OM, Nwoha PU, Olaibi OK (2012). Neuroprotective effects of kolaviron, a biflavonoid complex of *Garcinia kola*, on rats hippocampus against methamphetamine-induced neurotoxicity. Maced J Med Sci.

[CR117] Ikpesu TO, Tongo I, Ariyo A (2014). Restorative prospective of powdered seeds extract of *Garcinia kola* in *Chrysichthys furcatus* induced with glyphosate formulation. Chin J Biol.

[CR118] Ishola IO, Chaturvedi JP, Rai S (2013). Evaluation of amentoflavone isolated from Cnestis ferruginea Vahl ex DC (Connaraceae) on production of inflammatory mediators in LPS stimulated rat astrocytoma cell line (C6) and THP-1 cells. J Ethnopharmacol.

[CR119] Ishola IO, Adamson FM, Adeyemi OO (2017). Ameliorative effect of kolaviron, a biflavonoid complex from *Garcinia kola* seeds against scopolamine-induced memory impairment in rats: role of antioxidant defense system. Metab Brain Dis.

[CR120] Ito C, Itoigawa M, Miyamoto Y (1999). A new biflavonoid from *Calophyllum panciflorum* with antitumor-promoting activity. J Nat Prod.

[CR121] Iwu M, Igboko O (1982). Flavonoids of *Garcinia kola* seeds. J Nat Prod.

[CR122] Iwu MM, Igboko OA, Elekwa OK, Tempesta MS (1990). Prevention of thioacetamide-induced hepatotoxicity by biflavanones of *Garcinia kola*. Phytother Res.

[CR123] Iwu MM, Igboko OA, Okunji CO, Tempesta MS (1990). Antidiabetic and aldose reductase activities of biflavanones of *Garcinia kola*. J Pharm Pharmacol.

[CR124] Iwu MM, Igboko OA, Tempesta MS (1990). Biflavonoid constituents of *Garcinia kola* roots. Fitoterapia.

[CR125] Iwu M, Duncan C, Okunji CO (1999). Perspectives on new crops and new uses.

[CR126] Iwu MM, Diop AD, Meserole L, Okunji CO, Iwu MM, Wootton J (2002). *Garcinia kola*: a new look at an old adaptogenic agent. Advances in phytomedicine.

[CR127] Jackson DN, Yang L, Wu S (2015). Garcinia xanthochymus benzophenones promote hyphal apoptosis and potentiate activity of fluconazole against *Candida albicans* biofilms. Antimicrob Agents Chemother.

[CR128] Jamila N, Khairuddean M, Khan SN, Khan N (2014). Complete NMR assignments of bioactive rotameric (3→8) biflavonoids from the bark of *Garcinia hombroniana*. Magn Reson Chem.

[CR129] Jeffers V, Gao H, Checkley LA (2016). Garcinol inhibits GCN5-mediated lysine acetyltransferase activity and prevents replication of the parasite *Toxoplasma gondii*. Antimicrob Agents Chemother.

[CR130] Jouda J-B, Tamokou J-D, Mbazoa CD (2016). Antibacterial and cytotoxic cytochalasins from the endophytic fungus *Phomopsis* sp. harbored in *Garcinia kola* (Heckel) nut. BMC Complement Altern Med.

[CR131] Kabangu K, Galeffi C, Aonzo E (1987). A new biflavanone from the bark of *Garcinia kola*. Planta Med.

[CR132] Kalgutkar AS, Crews BC, Rowlinson SW (2000). Biochemically based design of cyclooxygenase-2 (COX-2) inhibitors: facile conversion of nonsteroidal antiinflammatory drugs to potent and highly selective COX-2 inhibitors. Proc Natl Acad Sci U S A.

[CR133] Kalu WO, Okafor PN, Ijeh II, Eleazu C (2016). Effect of kolaviron, a biflavanoid complex from *Garcinia kola* on some biochemical parameters in experimentally induced benign prostatic hyperplasic rats. Biomed Pharmacother.

[CR134] Kelly TR, Szabados A, Lee Y-J (1997). Total synthesis of garcifuran B. J Org Chem.

[CR135] Kilpatrick IC, Traut M, Heal DJ (2001). Monoamine oxidase inhibition is unlikely to be relevant to the risks associated with phentermine and fenfluramine: a comparison with their abilities to evoke monoamine release. Int J Obes.

[CR136] Kim HK, Son KH, Chang HW (1998). Amentoflavone, a plant biflavone: a new potential anti-inflammatory agent. Arch Pharm Res.

[CR137] Klein EA, Thompson IM, Tangen CM (2011). Vitamin E and the risk of prostate cancer: the selenium and vitamin E cancer prevention trial (SELECT). J Am Med Assoc.

[CR138] Klein-Júnior LC, Campos A, Niero R (2020). Xanthones and cancer: from natural sources to mechanisms of action. Chem Biodivers.

[CR139] Kluge S, Schubert M, Schmölz L, Ur Rahman A (2016). Garcinoic acid: a promising bioactive natural product for better understanding the physiological functions of tocopherol metabolites. Studies in natural products chemistry.

[CR140] Konziase B (2015). Protective activity of biflavanones from *Garcinia kola* against plasmodium infection. J Ethnopharmacol.

[CR141] Kopytko P, Piotrowska K, Janisiak J, Tarnowski M (2021). Garcinol—a natural histone acetyltransferase inhibitor and new anti-cancer epigenetic drug. Int J Mol Sci.

[CR142] Lau OD, Kundu TK, Soccio RE (2000). HATs off: Selective synthetic inhibitors of the histone acetyltransferases p300 and PCAF. Mol Cell.

[CR143] Lee JS, Sul JY, Park JB (2013). Fatty acid synthase inhibition by amentoflavone suppresses HER2/neu (erbB2) oncogene in SKBR3 human breast cancer cells. Phytother Res.

[CR144] Lee P-S, Teng C-Y, Kalyanam N (2019). Garcinol reduces obesity in high-fat-diet-fed mice by modulating gut microbiota composition. Mol Nutr Food Res.

[CR145] Lee P-S, Nagabhushanam K, Ho C-T, Pan M-H (2021). Inhibitory effect of garcinol on obesity-exacerbated, colitis-mediated colon carcinogenesis. Mol Nutr Food Res.

[CR146] Li F, Shanmugam MK, Chen L (2013). Garcinol, a polyisoprenylated benzophenone modulates multiple proinfl ammatory signaling cascades leading to the suppression of growth and survival of head and neck carcinoma. Cancer Prev Res (phila Pa).

[CR147] Li X, Ai H, Sun D (2016). Anti-tumoral activity of native compound morelloflavone in glioma. Oncol Lett.

[CR148] Lin Y-M, Anderson H, Flavin MT (1997). In vitro anti-HIV activity of biflavonoids isolated from Rhus succedanea and Garcinia multiflora. J Nat Prod.

[CR149] Lin S-H, Huang K-J, Weng C-F, Shiuan D (2015). Exploration of natural product ingredients as inhibitors of human HMG-CoA reductase through structure-based virtual screening. Drug Des Devel Ther.

[CR150] Liu C, Ho PCL, Wong FC (2015). Garcinol: Current status of its anti-oxidative, anti-inflammatory and anti-cancer effects. Cancer Lett.

[CR151] Liu S, Yang X, Zhang H (2020). Amentoflavone attenuates *Clostridium perfringens* gas gangrene by targeting alpha-toxin and Perfringolysin O. Front Pharmacol.

[CR152] Lobstein-Guth A, Briancon-Scheid F, Victoire C (1988). Isolation of amentoflavone from *Ginkgo biloba*. Planta Med.

[CR153] Luzzi R, Guimarães CL, Verdi LG (1997). Isolation of biflavonoids with analgesic activity from *Rheedia gardneriana* leaves. Phytomedicine.

[CR154] Lv X, Zhang J-B, Wang X-X (2018). Amentoflavone is a potent broad-spectrum inhibitor of human UDP-glucuronosyltransferases. Chem Biol Interact.

[CR155] Lyseng-Williamson KA, Perry CM (2003). Micronised purified flavonoid fraction: a review of its use in chronic venous insufficiency, venous ulcers and haemorrhoids. Drugs.

[CR156] Madubunyi II (1995). Antimicrobial activities of the constituents of *Garcinia kola* seeds. Int J Pharmacogn.

[CR157] Maňourová A, Leuner O, Tchoundjeu Z (2019). Medicinal potential, utilization and domestication status of bitter kola (*Garcinia kola* Heckel) in West and Central Africa. Forests.

[CR158] Mantelingu K, Reddy BAA, Swaminathan V (2007). Specific inhibition of p300-HAT alters global gene expression and represses HIV replication. Chem Biol.

[CR159] Marinelli R, Torquato P, Bartolini D (2020). Garcinoic acid prevents β-amyloid (Aβ) deposition in the mouse brain. J Biol Chem.

[CR160] Mazumder MK, Bhattacharjee N, Borah A (2016). Garcinol prevents hyperhomocysteinemia and enhances bioavailability of L-DOPA by inhibiting catechol-O-methyltransferase: an in silico approach. Med Chem Res.

[CR161] Mazumder MK, Paul R, Phukan BC (2018). Garcinol, an effective monoamine oxidase-B inhibitor for the treatment of Parkinson’s disease. Med Hypotheses.

[CR162] Mazzini F, Betti M, Netscher T (2009). Configuration of the vitamin E analogue garcinoic acid extracted from *Garcinia Kola* seeds. Chirality.

[CR163] Mbwambo ZH, Kapingu MC, Moshi MJ (2006). Antiparasitic activity of some xanthones and biflavonoids from the root bark of Garcinia livingstonei. J Nat Prod.

[CR164] Miladiyah I, Jumina J, Haryana SM, Mustofa M (2018). Biological activity, quantitative structure-activity relationship analysis, and molecular docking of xanthone derivatives as anticancer drugs. Drug Des Devel Ther.

[CR165] Miller WR, Furr BJA (2006). Background and development of aromatase inhibitors. Aromatase inhibitors.

[CR166] Monago CC, Akhidue V (2002). Estimation of tannin, saponin, oxalate, cyanogenic and cardiac glycosides in *Garsinia kola*. J Appl Sci Environ Manag.

[CR167] Monsey MS, Sanchez H, Taylor JR (2017). The naturally occurring compound *Garcinia indica* selectively impairs the reconsolidation of a cocaine-associated memory. Neuropsychopharmacology.

[CR168] Mountessou BYG, Tchamgoue J, Paul Dzoyem J (2018). Two xanthones and two rotameric (3 → 8) biflavonoids from the Cameroonian medicinal plant *Allanblackia floribunda* Oliv. (Guttiferae). Tetrahedron Lett.

[CR169] Na M, Kim KA, Oh H (2007). Protein tyrosine phosphatase 1B inhibitory activity of amentoflavone and its cellular effect on tyrosine phosphorylation of insulin receptors. Biol Pharm Bull.

[CR170] Negi PS, Jayaprakasha GK (2004). Control of foodborne pathogenic and spoilage bacteria by garcinol and garcinia indicaextracts, and their antioxidant activity. J Food Sci.

[CR171] Niwa M, Terashima K, Aqil M (1993). Garcinol, a novel arylbenzofuran derivative from *Garcinia kola*. Heterocycles.

[CR172] Niwa M, Ito J, Terashima K, Aqil M (1994). Garcipyran, a novel 6-aryl-1,2-benzopyran derivative from *Garcinia kola*. Heterocycles.

[CR173] Niwa M, Terashima K, Ito J, Aqil M (1994). Two novel arylbenzofurans, garcifuran-A and garcifuran-B from *Garcinia kola*. Heterocycles.

[CR174] Nokhala A, Siddiqui MJ, Ahmed QU (2020). Investigation of α-glucosidase inhibitory metabolites from Tetracera scandens leaves by GC–MS metabolite profiling and docking studies. Biomolecules.

[CR175] Nworu CS, Akah PA, Esimone CO (2008). Immunomodulatory activities of kolaviron, a mixture of three related biflavonoids of *Garcinia kola* Heckel. Immunopharmacol Immunotoxicol.

[CR176] Offor U, Ajayi SA, Jegede IA (2017). Renal histoarchitectural changes in nevirapine therapy: possible role of kolaviron and vitamin C in an experimental animal model. Afr Health Sci.

[CR177] Oike T, Ogiwara H, Torikai K (2012). Garcinol, a histone acetyltransferase inhibitor, radiosensitizes cancer cells by inhibiting non-homologous end joining. Int J Radiat Oncol Biol Phys.

[CR178] Ojo OA, Ojo AB, Maimako RF (2021). Exploring the potentials of some compounds from Garcinia kola seeds towards identification of novel PDE-5 inhibitors in erectile dysfunction therapy. Andrologia.

[CR179] Okafor AI, Onyike E (2020). Inhibition of key enzymes linked to snake venom induced local tissue damage by kolaviron. J Basic Clin Physiol Pharmacol.

[CR180] Okoko T (2009). In vitro antioxidant and free radical scavenging activities of *Garcinia kola* seeds. Food Chem Toxicol.

[CR181] Okoko T (2018). Kolaviron and selenium reduce hydrogen peroxide-induced alterations of the inflammatory response. J Genet Eng Biotechnol.

[CR182] Okoko T, Ndoni SA (2021). Protective effect of kolaviron on bromate-induced toxicity on raw u937 cells and macrophages. Malays J Biochem Mol Biol.

[CR183] Okoye TC, Uzor PF, Onyeto CA, Okerere EK, Kuete V (2014). Safe African medicinal plants for clinical studies. Toxicological survey of African medicinal plants.

[CR184] Okwu DE (2005). Phytochemicals, vitamins and mineral contents of two Nigerian medicinal plants. Int J Mol Med Adv Sci.

[CR185] Ola OS, Adewole KE (2021). Anticlastogenic and hepatoprotective effects of Kolaviron on sodium valproate-induced oxidative toxicity in Wistar rats. Egypt J Basic Appl Sci.

[CR186] Olajide OJ, Enaibe BU, Bankole OO (2015). Kolaviron was protective against sodium azide (NaN3) induced oxidative stress in the prefrontal cortex. Metab Brain Dis.

[CR187] Olajide OJ, Akinola BO, Ajao SM, Enaibe BU (2016). Sodium azide-induced degenerative changes in the dorsolateral prefrontal cortex of rats: attenuating mechanisms of kolaviron. Eur J Anat.

[CR188] Olajide OA, Iwuanyanwu VU, Lepiarz-Raba I (2021). *Garcinia kola* and garcinoic acid suppress SARS-CoV-2 spike glycoprotein S1-induced hyper-inflammation in human PBMCs through inhibition of NF-κB activation. Phytother Res.

[CR189] Olaleye SB, Farombi EO (2006). Attenuation of indomethacin- and HCl/ethanol-induced oxidative gastric mucosa damage in rats by kolaviron, a natural biflavonoid of *Garcinia kola* seed. Phytother Res.

[CR190] Olaleye SB, Onasanwo SA, Ige AO (2010). Anti-inflammatory activities of a kolaviron-inhibition of nitric oxide, prostaglandin E2 and tumor necrosis factor-alpha production in activated macrophage-like cell line. Afr J Med Med Sci.

[CR191] Olatoye FJ, Akindele AJ, Onwe S (2021). Ameliorative effect of Kolaviron, an extract of *Garcinia kola* seeds, on induced hypertension. J Complement Integr Med.

[CR192] Olatunde Farombi E (2000). Mechanisms for the hepatoprotective action of kolaviron: studies on hepatic enzymes, microsomal lipids and lipid peroxidation in carbontetrachloride-treated rats. Pharmacol Res.

[CR193] Oluwatosin A, Tolulope A, Ayokulehin K (2014). Antimalarial potential of kolaviron, a biflavonoid from *Garcinia kola* seeds, against *Plasmodium berghei* infection in Swiss albino mice. Asian Pac J Trop Med.

[CR194] Omole JG, Ayoka OA, Alabi QK (2018). Protective effect of kolaviron on cyclophosphamide-induced cardiac toxicity in rats. J Evid-Based Integr Med.

[CR195] Omotoso GO, Olajide OJ, Gbadamosi IT (2018). Kolaviron protects the prefrontal cortex and hippocampus against histomorphological and neurobehavioural changes in cuprizone model of multiple sclerosis. Malays J Med Sci.

[CR196] Omotoso GO, Ukwubile II, Arietarhire L (2018). Kolaviron protects the brain in cuprizone-induced model of experimental multiple sclerosis via enhancement of intrinsic antioxidant mechanisms: Possible therapeutic applications?. Pathophysiology.

[CR197] Omotoso GO, Olajide OJ, Gbadamosi IT (2019). Cuprizone toxicity and Garcinia kola biflavonoid complex activity on hippocampal morphology and neurobehaviour. Heliyon.

[CR198] Omotoso GO, Arietarhire LO, Ukwubile II, Gbadamosi IT (2020). Research paper: the protective effect of kolaviron on molecular, cellular, and behavioral characterization of cerebellum in the rat model of demyelinating diseases. Basic Clin Neurosci.

[CR199] Omotoso GO, Mutholib NY, Abdulsalam FA, Bature AI (2020). Kolaviron protects against cognitive deficits and cortico-hippocampal perturbations associated with maternal deprivation in rats. Anat Cell Biol.

[CR200] Onasanwo SA, Singh N, Olaleye SB, Palit G (2011). Anti-ulcerogenic and proton pump (H+, K+ ATPase) inhibitory activity of Kolaviron from *Garcinia kola* Heckel in rodents. Indian J Exp Biol.

[CR201] Onyekwelu JC, Oyewale O, Stimm B, Mosandl R (2015). Antioxidant, nutritional and anti-nutritional composition of *Garcinia kola* and *Chrysophyllum albidum* from rainforest ecosystem of Ondo State, Nigeria. J For Res.

[CR202] Owoeye O, Adedara IA, Bakare OS (2014). Kolaviron and vitamin e ameliorate hematotoxicity and oxidative stress in brains of prepubertal rats treated with an anticonvulsant phenytoin. Toxicol Mech Methods.

[CR203] Owoeye O, Adedara IA, Adeyemo OA (2015). Modulatory role of kolaviron in phenytoin-induced hepatic and testicular dysfunctions in wistar rats. J Diet Suppl.

[CR204] Oyagbemi A, Omobowale T, Farombi E (2016). Kolaviron and *Garcinia kola* attenuate homocysteine-induced arteriosclerosis and cardiotoxicity in Wistar rats. Toxicol Int.

[CR205] Oyagbemi AA, Bester D, Esterhuyse J, Farombi EO (2017). Kolaviron, a biflavonoid of *Garcinia kola* seed mitigates ischemic/reperfusion injury by modulation of pro-survival and apoptotic signaling pathways. J Intercult Ethnopharmacol.

[CR206] Oyagbemi AA, Bester D, Esterhuyse J, Farombi EO (2018). Kolaviron and *Garcinia kola* seed extract protect against ischaemia/reperfusion injury on isolated rat heart. Drug Res.

[CR207] Oyagbemi AA, Omobowale TO, Asenuga ER (2018). Kolaviron attenuated arsenic acid induced-cardiorenal dysfunction via regulation of ROS, C-reactive proteins (CRP), cardiac troponin I (CTnI) and BCL2. J Tradit Complement Med.

[CR208] Oyagbemi AA, Omobowale TO, Olopade JO, Farombi EO (2018). Kolaviron and *Garcinia kola* attenuate doxorubicin-induced cardiotoxicity in Wistar rats. J Complement Integr Med.

[CR209] Oyenihi OR, Brooks NL, Oguntibeju OO (2015). Effects of kolaviron on hepatic oxidative stress in streptozotocin induced diabetes. BMC Complement Altern Med.

[CR210] Oyenihi OR, Cerf ME, Matsabisa MG (2021). Effect of kolaviron on islet dynamics in diabetic rats. Saudi J Biol Sci.

[CR211] Oyovwi MO, Ben-Azu B, Edesiri TP (2021). Kolaviron abates busulfan-induced episodic memory deficit and testicular dysfunction in rats: the implications for neuroendopathobiological changes during chemotherapy. Biomed Pharmacother.

[CR212] Pan M-H, Chang W-L, Lin-Shiau S-Y (2001). Induction of apoptosis by garcinol and curcumin through cytochrome c release and activation of caspases in human leukemia HL-60 cells. J Agric Food Chem.

[CR213] Pang X, Yi T, Yi Z (2009). Morelloflavone, a biflavonoid, inhibits tumor angiogenesis by targeting Rho GTPases and extracellular signal-regulated kinase signaling pathways. Cancer Res.

[CR214] Park S-Y, Nguyen P-H, Kim G (2020). Strong and selective inhibitory effects of the biflavonoid selamariscina a against CYP2C8 and CYP2C9 enzyme activities in human liver microsomes. Pharmaceutics.

[CR215] Pinkaew D, Cho SG, Hui DY (2009). Morelloflavone blocks injury-induced neointimal formation by inhibiting vascular smooth muscle cell migration. Biochim Biophys Acta Gen Subj.

[CR216] Pinkaew D, Hutadilok-Towatana N, Teng B-B (2012). Morelloflavone, a biflavonoid inhibitor of migration-related kinases, ameliorates atherosclerosis in mice. Am J Physiol Heart Circ Physiol.

[CR217] Qin L, Zhao Y, Zhang B, Li Y (2018). Amentoflavone improves cardiovascular dysfunction and metabolic abnormalities in high fructose and fat diet-fed rats. Food Funct.

[CR218] Raikar SB, Nuhant P, Delpech B, Marazano C (2008). Synthesis of polyprenylated benzoylphloroglucinols by regioselective prenylation of phloroglucinol in an aqueous medium. Eur J Org Chem.

[CR219] Ramakrishnan S, Paramewaran S, Nasir NM (2021). Synthetic approaches to biologically active xanthones: an update. Chem Pap.

[CR220] Ranjbarnejad T, Saidijam M, Tafakh MS (2017). Garcinol exhibits anti-proliferative activities by targeting microsomal prostaglandin e synthase-1 in human colon cancer cells. Hum Exp Toxicol.

[CR221] Recalde-Gil MA, Klein-Júnior LC, Dos Santos PC (2017). Monoamine oxidase inhibitory activity of biflavonoids from branches of *Garcinia gardneriana* (Clusiaceae). Nat Prod Commun.

[CR222] Recalde-Gil AM, Klein-Júnior L, Salton J (2019). Aromatase (CYP19) inhibition by biflavonoids obtained from the branches of *Garcinia gardneriana* (Clusiaceae). Z Naturforschung Sect C J Biosci.

[CR223] Rizk YS, Santos-Pereira S, Gervazoni L (2021). Amentoflavone as an ally in the treatment of cutaneous leishmaniasis: analysis of its antioxidant/prooxidant mechanisms. Front Cell Infect Microbiol.

[CR224] Robinson I, De Serna DG, Gutierrez A, Schade DS (2006). Vitamin E in humans: an explanation of clinical trial failure. Endocr Pract.

[CR225] Rubin JE, Ball KR, Chirino-Trejo M (2011). Antimicrobial susceptibility of *Staphylococcus aureus* and *Staphylococcus pseudintermedius* isolated from various animals. Can Vet J.

[CR226] Ryu YB, Jeong HJ, Kim JH (2010). Biflavonoids from *Torreya nucifera* displaying SARS-CoV 3CLpro inhibition. Bioorg Med Chem.

[CR227] Ryu Y-K, Park H-Y, Go J (2018). Effects of histone acetyltransferase inhibitors on l-DOPA-induced dyskinesia in a murine model of Parkinson’s disease. J Neural Transm.

[CR228] Saadat N, Akhtar S, Goja A (2018). Dietary garcinol arrests pancreatic cancer in p53 and K-ras conditional mutant mouse model. Nutr Cancer.

[CR229] Sabogal-Guáqueta AM, Carrillo-Hormaza L, Osorio E, Cardona-Gómez GP (2018). Effects of biflavonoids from Garcinia madruno on a triple transgenic mouse model of Alzheimer’s disease. Pharmacol Res.

[CR230] Sadasivan Nair AN, Raveendran Nair RV, Rajendran Nair AP (2020). Antidiabetes constituents, cycloartenol and 24-methylenecycloartanol, from ficus krishnae. PLoS ONE.

[CR231] Saenz De Tejada I, Angulo J, Cuevas P (2001). The phosphodiesterase inhibitory selectivity and the in vitro and in vivo potency of the new PDE5 inhibitor vardenafil. Int J Impot Res.

[CR232] Sakthivel KM, Guruvayoorappan C (2013). Amentoflavone inhibits iNOS, COX-2 expression and modulates cytokine profile, NF-κB signal transduction pathways in rats with ulcerative colitis. Int Immunopharmacol.

[CR233] Salau VF, Erukainure OL, Koorbanally NA, Islam MS (2020). Kolaviron modulates dysregulated metabolism in oxidative pancreatic injury and inhibits intestinal glucose absorption with concomitant stimulation of muscle glucose uptake. Arch Physiol Biochem.

[CR234] Salau VF, Erukainure OL, Bharuth V (2021). Kolaviron stimulates glucose uptake with concomitant modulation of metabolic activities implicated in neurodegeneration in isolated rat brain, without perturbation of tissue ultrastructural morphology. Neurosci Res.

[CR235] Salehi B, Mishra AP, Nigam M (2018). Resveratrol: a double-edged sword in health benefits. Biomedicines.

[CR236] Saponara R, Bosisio E (1998). Inhibition of cAMP-phosphodiesterase by biflavones of *Ginkgo biloba* in rat adipose tissue. J Nat Prod.

[CR237] Schobert R, Biersack B (2019). Chemical and biological aspects of garcinol and isogarcinol: recent developments. Chem Biodivers.

[CR238] Sesso HD, Buring JE, Christen WG (2008). Vitamins E and C in the prevention of cardiovascular disease in men: the physicians’ health study II randomized controlled trial. J Am Med Assoc.

[CR239] Shin DH, Bae YC, Kim-Han JS (2006). Polyphenol amentoflavone affords neuroprotection against neonatal hypoxic-ischemic brain damage via multiple mechanisms. J Neurochem.

[CR240] Steinhubl SR (2008). Why have antioxidants failed in clinical trials?. Am J Cardiol.

[CR241] Su C, Yang C, Gong M (2019). Antidiabetic activity and potential mechanism of amentoflavone in diabetic mice. Molecules.

[CR242] Tabares-Guevara JH, Lara-Guzmán OJ, Londoño-Londoño JA (2017). Natural biflavonoids modulate macrophage-oxidized LDL interaction in vitro and promote atheroprotection in vivo. Front Immunol.

[CR243] Tauchen J, Huml L, Rimpelova S, Jurášek M (2020). Flavonoids and related members of the aromatic polyketide group in human health and disease: do they really work?. Molecules.

[CR244] Tcheghebe OT, Signe M, Seukep AJ, Tatong FN (2016). Review on traditional uses, phytochemical and pharmacological profiles of *Garcinia kola* Heckel. J Med Med Sci.

[CR245] Tchimene KM, Anaga AO, Ugwoke CEC (2015). Bio-flavonoids and garcinoic acid from *Garcinia kola* seeds with promising anti-inflammatory potentials. Pharmacogn J.

[CR246] Tchimene MK, Aanaga AO, Ugwoke CEC (2015). Bio-flavonoids and garcinoic acid from *Garcinia kola* seeds with promising local anesthetic potentials. Int J Pharmacogn Phytochem Res.

[CR247] Teodoro JS, Machado IF, Castela AC, Gupta RC, Lall R, Srivastava A (2021). Mitochondria as a target for safety and toxicity evaluation of nutraceuticals. Nutraceuticals: efficacy, safety and toxicity.

[CR271] Terashima K, Aqil M, Niwa M (1995). Garcinianin, a novel biflavonoid from the roots of *Garcinia kola*. Heterocycles.

[CR248] Terashima K, Shimamura T, Tanabayashi M (1997). Constituents of the seeds of *Garcinia kola*: two new antioxidants, garcinoic acid and garcinal. Heterocycles.

[CR249] Terashima K, Kondo Y, Aqil M, Niwa M (1999). A new xanthone from the stems of *Garcinia kola*. Nat Prod Lett.

[CR250] Terashima K, Takaya Y, Niwa M (2002). Powerful antioxidative agents based on garcinoic acid from *Garcinia kola*. Bioorg Med Chem.

[CR251] Timothy MR, Ibrahim YKE, Muhammad A (2021). Trypanosuppressive effects of kolaviron may be associated with down regulation of trypanothione reductase in trypanosoma congolense infection. Trop Biomed.

[CR252] Trisuwan K, Rukachaisirikul V, Phongpaichit S, Hutadilok-Towatana N (2013). Tetraoxygenated xanthones and biflavanoids from the twigs of *Garcinia merguensis*. Phytochem Lett.

[CR253] Tshibangu PT, Kapepula PM, Kapinga MJK (2016). Fingerprinting and validation of a LC-DAD method for the analysis of biflavanones in *Garcinia kola* -based antimalarial improved traditional medicines. J Pharm Biomed Anal.

[CR254] Tu S-H, Chiou Y-S, Kalyanam N (2017). Garcinol sensitizes breast cancer cells to Taxol through the suppression of caspase-3/iPLA2 and NF-κB/Twist1 signaling pathways in a mouse 4T1 breast tumor model. Food Funct.

[CR255] Tuansulong K-A, Hutadilok-Towatana N, Mahabusarakam W (2011). Morelloflavone from *Garcinia dulcis* as a novel biflavonoid inhibitor of HMG-CoA reductase. Phytother Res.

[CR256] Uche OK, Osakpolo RFA (2018). Kolaviron attenuates elevation in blood pressure and ameliorates dyslipidemia in salt-induced hypertensive sprague-dawley rats. Afr J Biomed Res.

[CR257] Uko OJ, Usman A, Ataja AM (2001). Some biological activities of *Garcinia kola* in growing rats. Vet Arh.

[CR258] Uwagie-Ero EA, Nwaehujor CO, Ode JO (2020). Investigation of polyisoprenyl benzophenone for anti-ulcer potentials in ethanol-hcl-induced gastric ulcerations in albino rats. Trop J Nat Prod Res.

[CR259] Wallert M, Bauer J, Kluge S (2019). The vitamin E derivative garcinoic acid from *Garcinia kola* nut seeds attenuates the inflammatory response. Redox Biol.

[CR260] Wang Y, Tsai M-L, Chiou L-Y (2015). Antitumor activity of garcinol in human prostate cancer cells and xenograft mice. J Agric Food Chem.

[CR261] Wang B, Lin L, Ai Q (2016). HAT inhibitor, garcinol, exacerbates lipopolysaccharide-induced inflammation in vitro and in vivo. Mol Med Rep.

[CR262] Wang Y-W, Zhang X, Chen C-L (2017). Protective effects of Garcinol against neuropathic pain: evidence from in vivo and in vitro studies. Neurosci Lett.

[CR263] Wilsky S, Sobotta K, Wiesener N (2012). Inhibition of fatty acid synthase by amentoflavone reduces coxsackievirus B3 replication. Arch Virol.

[CR264] Winner K, Polycarp O, Ifeoma I, Chinedum E (2016). Effect of fractions of kolaviron on some indices of benign prostatic hyperplasia in rats: identification of the constituents of the bioactive fraction using GC-MS. RSC Adv.

[CR265] Xiong X, Tang N, Lai X (2021). Insights into amentoflavone: a natural multifunctional biflavonoid. Front Pharmacol.

[CR266] Yu S, Yan H, Zhang L (2017). A review on the phytochemistry, pharmacology, and pharmacokinetics of amentoflavone, a naturally-occurring biflavonoid. Molecules.

[CR267] Zhao X, Liu B, Liu S (2017). Anticytotoxin effects of amentoflavone to pneumolysin. Biol Pharm Bull.

[CR268] Zhao J, Yang T, Ji J (2018). Garcinol exerts anti-cancer effect in human cervical cancer cells through upregulation of T-cadherin. Biomed Pharmacother.

[CR269] Zhao N, Sun C, Zheng M (2019). Amentoflavone suppresses amyloid β1–42 neurotoxicity in Alzheimer’s disease through the inhibition of pyroptosis. Life Sci.

[CR270] Zhuang J-L, Liu Y-Y, Li Z-Z (2021). Amentoflavone prevents ox-LDL-induced lipid accumulation by suppressing the PPARγ/CD36 signal pathway. Toxicol Appl Pharmacol.

